# Effects of vegetation, terrain and soil layer depth on eight soil chemical properties and soil fertility based on hybrid methods at urban forest scale in a typical loess hilly region of China

**DOI:** 10.1371/journal.pone.0205661

**Published:** 2018-10-18

**Authors:** Xinping Zhang, Fangfang Zhang, Dexiang Wang, Junxi Fan, Youning Hu, Haibin Kang, Mingjie Chang, Yue Pang, Yang Yang, Yang Feng

**Affiliations:** 1 College of Forestry, Northwest A&F University, Yangling, China; 2 Tourism Department, Shaanxi Vocational & Technical College, Xi’an, China; 3 College of Arts and Design, Xi’an University of Technology, Xi’an, China; 4 Branch School of Gaoling District Xi’an City, Shaanxi Agricultural Broadcasting and Television School, Xi’an, China; 5 College of Landscape Architecture and Arts, Northwest A&F University, Yangling, China; 6 School of Biological and Environmental Engineering, Xi’an University, Xi’an, China; Fred Hutchinson Cancer Research Center, UNITED STATES

## Abstract

Although the spatial mapping and fertility assessment of soil chemical properties (SCPs) are well studied in the Loess Plateau region of China at farmland scale, little is known about spatial mapping the SCPs and their fertility and their influence factors at urban forest scale. The objectives of this study were to (1) compare the performance of two spatial interpolation methods, Ordinary kriging (OK) and regression kriging (RK), and (2) explain the relationships of the vegetation, terrain, and soil layer depth between the eight SCPs and their fertility, and (3) find the limiting factors of soil comprehensive fertility in this study area? The Yan’an urban forest was taken as study case, used hybrid spatial interpolation methods based on OK and RK to mapping eight SCPs and the soil fertility in each soil layer (0–20 cm, 20–40 cm, and 40–60 cm) for 285 soil samples. The results indicated that RK outperformed OK for total nitrogen (TN), available potassium (AK), organic matter (OM) in 0–60 cm profile and available phosphorus (AP) in the 0–20 cm and 40–60 cm soil layers because RK considered the impact of terrain. The terrain factors, comprising the relative terrain position, slope, aspect, and relative elevation significantly affected the SCPs and spatial heterogeneity of fertility, where the vegetation cover types determined the average SCPs to some extent. On average, the six SCPs (except total potassium and AP) and the fertility decreased as the soil layer depth increased. Ten vegetation cover types comprising broadleaved mixed natural forest (BM), cultivated land (CL), economic forest (EF), grassland (GL), *Platycladus orientalis* natural forest (PON), *Platycladus orientalis* plantation (POP), *Pinus tabuliformis* plantation (PT), *Quercus wutaishanica* natural forest (QW), *Robinia pseudoacacia* plantation (RP), and Shrubwood (SW) were associated with significant differences in TN, OM, AN, AP, and AK, across the three soil layers. QW, PON, and BM also had higher content of TN, OM, AN, and AK contents than the other vegetation cover types. There were small differences in TK, AK, and pH among the 10 vegetation cover types. We concluded that AN, TN, and OM are the limiting factors of soil comprehensive fertility in this region. These results improve understanding of the spatial mapping, influence and limiting factors of SCPs and their fertility at urban forest scales.

## Introduction

Soil plays an essential role in the biosphere by governing plant productivity, organic matter (OM) degradation, and nutrient cycles [1]. Soil fertility is one of the major drivers of ecological processes, and thus it is frequently investigated in ecological research [2]. The eight soil chemical properties (SCPs), comprising total nitrogen (TN), total potassium (TK), total potassium (TP), available nitrogen (AN), available phosphorus (AP), available potassium (AK) and organic matter (OM) are important chemical components of soil fertility. Hence, spatially continuous mapping of these soil chemical properties and soil fertility is required to facilitate the sustainable management of land resources in precision agriculture and forestry [3–5], while it is also helpful to understand the belowground food webs [6], plant species distributions [7], and other factors. However, abundant observations of soil properties cannot always be obtained easily across a large landscape because of cost and time constraints on soil sampling and analysis [8]. Therefore, spatial interpolation is commonly used to generate soil property maps from discrete point-based data [9]. Previous studies have shown that auxiliary variables are important for predicting soil properties [10]. In recent years, the availability of high-resolution topographical data has provided ancillary variables for accurately mapping soil chemical properties [11–14]. The interpolation method employed critically affects the accuracy of interpolation. Among the existing interpolation techniques, the two commonly used methods are ordinary kriging (OK) [3,12,15,16] and regression kriging (RK) [8,16–19]. RK performs better than OK because its uses auxiliary variables, as well as reducing the number of observations needed for target variables [20]. Previous studies have investigated the effects of topography and the dominant trees species on the spatial distribution of soil physicochemical properties [3,12,21–23]. Terrain is one of the main factors that affect the soil C, N, and P contents at the landscape scale [3,21,24]. In recent decades, many studies have shown that a geostatistical approach based on the integration of terrain factors is an effective tool for accurately predicting the spatial distribution of soil chemical properties [3,15,21,25–29]. Fraterrigo et al. [30] showed that vegetation cover types have persistent, long-term effects on the spatial heterogeneity of soil resources, which may not be detectable when the values are equalized across sites. Differences in the distribution and supply of soil chemical properties could alter the composition and diversity of forest ecosystems by interacting with the patterns of variability in the plant and heterotrophic organisms. These activities may continue to influence the distributions of soil nutrients by altering their spatial heterogeneity in ecologically sensitive regions, such as, the Loess Plateau in China (LPC).

The LPC is well known due to the presence of severe soil erosion and ecosystem degradation, which have resulted in great soil nutrient losses and extreme terrain conditions [31–33]. Spatial information about soil nutrients and its fertility is required to understand and manage loess hilly ecosystems [24,34]. Previous studies in this area mainly focused on agricultural systems [35–37], whereas the continuous spatial distributions of these soil chemical properties and fertility, as well as the effects of the vegetation, terrain, and soil layer depth remain unclear in urban forest ecosystems. Thus, in order to determine the spatial distributions of the main soil chemical properties and their fertility, we investigated Yan’an urban forest as a real-world case study.

Urban forests are integral components of urban ecosystems and they can generate significant ecosystem services. Yan’an urban forest is coupled of artificial and natural forest ecosystem, which comprises suburban forest (including grassland), urban green spaces, and street trees. This region has been selected as one of the pioneer demonstration areas for the large-scale ecological restoration project known as “Grain for Green” in China in 1999 [38–41]. After ecological restoration for 15 years, the forest coverage of Yan’an urban forest increased significantly from 36.6% to 65.8%, and which effectively improved the ecological functions and services of Yan'an urban forest, such as offsetting carbon emissions, removing air pollutants, regulating the microclimate, allowing recreation [42], and mitigating urban heat islands [38]. The annual total value of these services is approximately 10.02 billion Chinese yuan [43].

In this study, in order to compare the performance of OK and RK in SCPs spatial predication, and to reveal the influence of vegetation, terrain and soil layer depth on eight soil chemical properties and soil fertility at urban forest scale, Ordinary Kriging (OK), Regression Kriging (RK), and an improved Nemerow index were used to mapping and assess soil chemical properties and their fertility, based on field vegetation investigations and the determination of soil chemical properties from 855 soil samples in 95 sample plots distributed evenly in the study area.

## Materials and methods

### Study area

**Fig 1:**
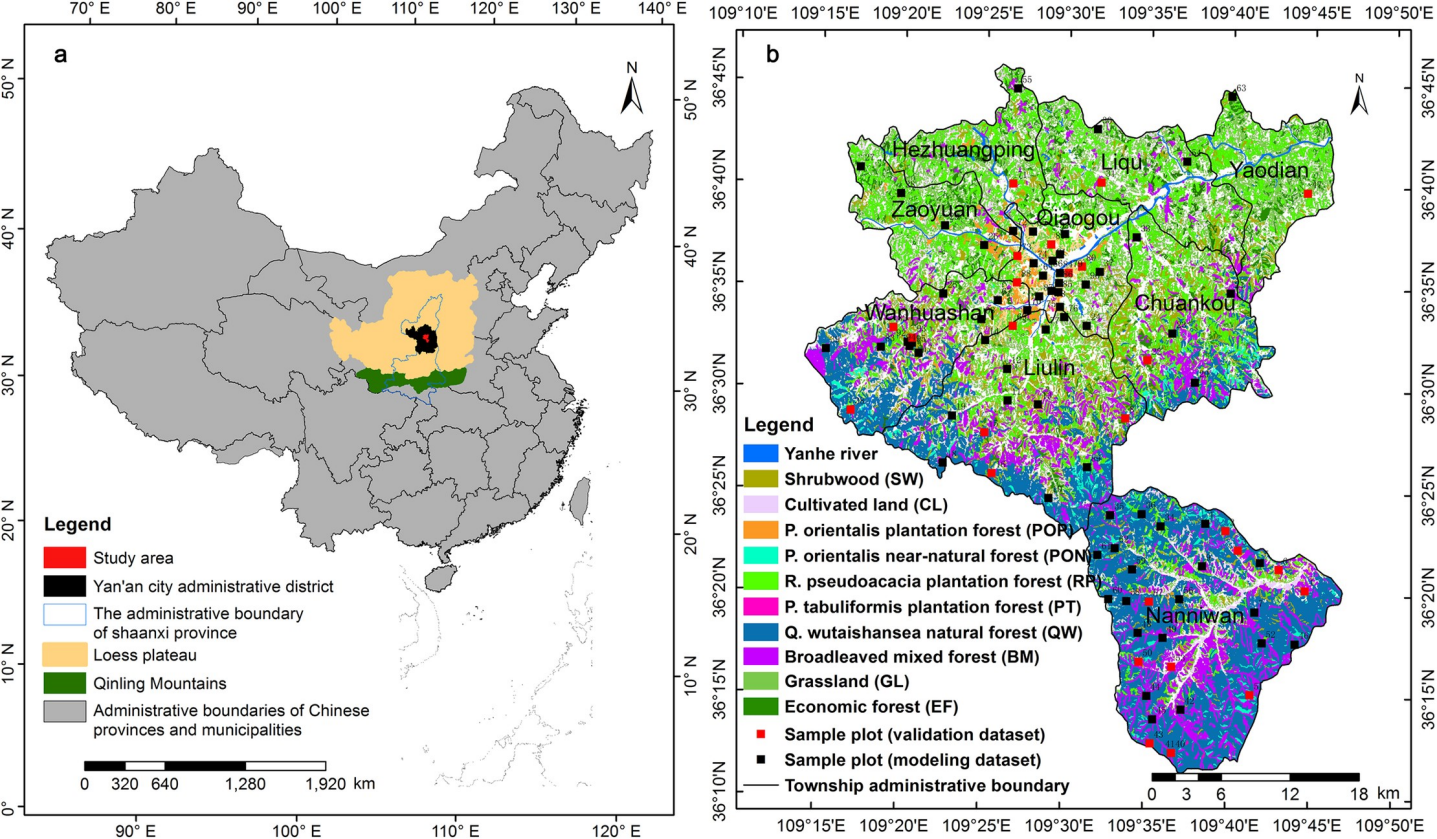
Maps of the study area showing: (a) its location in China and; (b) the spatial distribution of 95 sample plots (black filled squares: modeling dataset, *n* = 70; red filled squares: validation dataset, *n* = 25).

According to the concept of urban forest and the local forestry practice [38,43], nine towns near (within 30 km) the center of Yan’an city, in the north of Shaanxi province, China, namely, Zaoyuan, Qiaogou, Chuankou, Liqu, Liulin, Yaodian, Wanhuashan, Hezhuangping, Nanniwan were identified as the spatial range of Yan'an urban forest (109°11′ to 109°47′ N, 36°11′ to 36°46′ E, elevation = 815–1466 m, total area = 1545.05 km^2^), which located in the hilly and gully region of the Loess Plateau (Fig 1). The main characteristics of Yan'an urban forest are as follows: (a) By the end of 2015, the total area and coverage rate of forests and trees in the study area was 1016.64 km^2^ (suburb forests = 998.76 km^2^, urban green spaces = 16.73 km^2^, street trees = 1.15 km^2^) and 65.8%, respectively. (b) The soil parent material is relatively homogeneous, the soil type is mainly loessial soil and the thickness ranges from approximately 50 m to 200 m [44], the soil has been deposited by the wind since the Quaternary period [45]. (c) However, the water retention properties of loessial soil are very poor because of its low anti-scourability [43]. The landforms of this area exhibit significant topographic variability, which corresponds to the different developmental stages and patterns of the Loess Plateau, including river valley, loess tableland, loess ridge, loess hill and loess residual tableland [44,46]. The river valley areas are relatively flat and solid, hence, the overwhelming majority of residents and human activities are mainly concentrated in this region. Because the other landforms have strong ecological fragility, state and local forestry sectors had proposed a series of protective measures, such as “Close Hillsides to Facilitate Afforestation”, “Returning Sloping Cultivated Land to Forest and Grasslands”, “Tending of Existing Woodlands”, and “Grazing Prohibition for Existing Grasslands”, since 1999. Which have effectively controlled the disturbance to Yan’an urban forest, especially the soils. (d) This area has a typical semiarid continental climate with average annual rainfall of approximately 470 mm, over 65% occurs during June and September. (e) The northern region mainly consists of *Robinia pseudoacacia* plantation and *Platycladus orientalis* plantation, and a few of economic forests, i.e., apple (*Malus pumila*), walnut (*Juglans regia*), etc.. While the southern region mainly covered by *Quercus wutaishanica* natural forest. The dominant tree species in the study area were listed in the Supporting Information Section (S1 Table and S2 Table).

### Technical details

**Fig 2:**
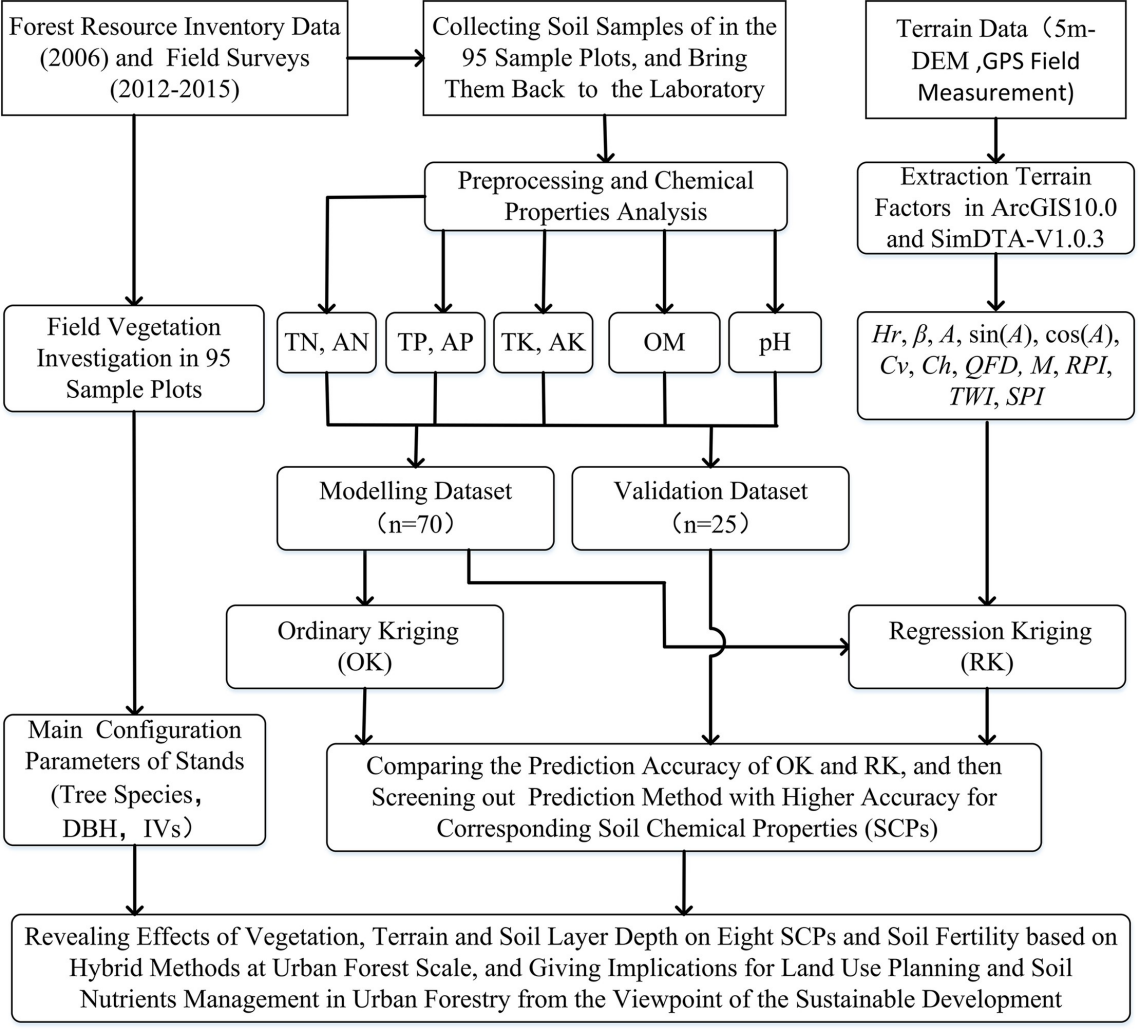
Flow chart illustrating the process followed in this study.

The workflow and technical details of this study are shown in Fig 2.

### Soil sampling and soil chemical analysis

**Fig 3:**
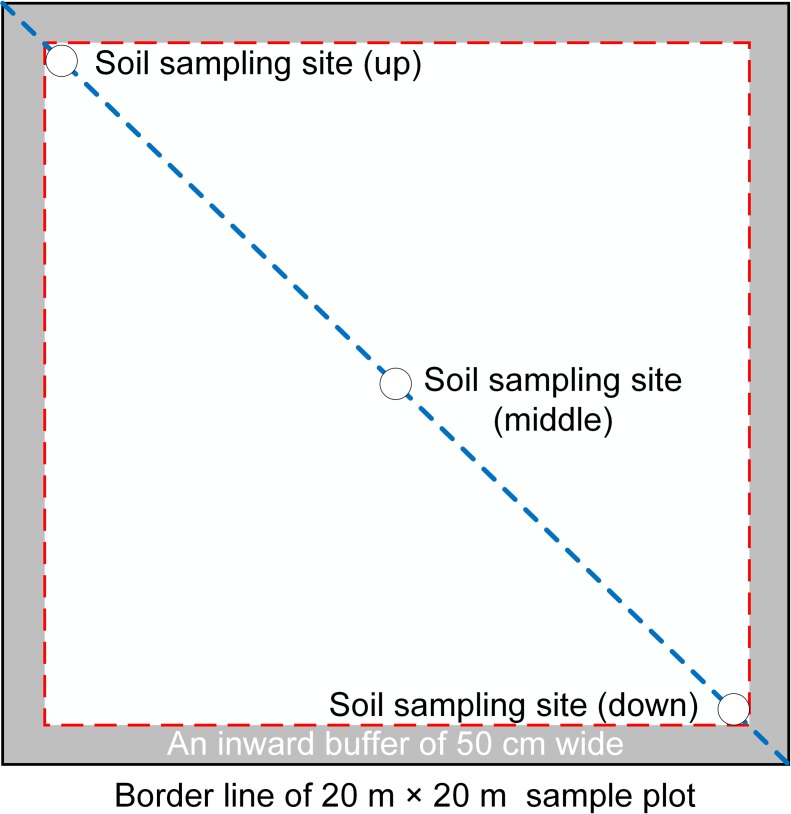
Schematic illustration of the soil sampling method.

In July and August during 2014 and 2015, 855 soil samples (95 sample plots × 3 layers × 3 positions) were collected from depths of 0 cm to 60 cm and divided into three layers, i.e., surface soils (0–20 cm depth), near-surface soils (20–40 cm depth), and subsurface soils (40–60 cm depth). Details of the 95 sample plots are shown in Table 1. We used a split core sampler with a diameter of 5 cm at each location after removing any litter. In each sample plot, we tested three soil sampling locations according to the slope position (up, middle, and down) along a diagonal path, where the soil samples from the three position were mixed by hand to obtain one homogeneous soil sample (Fig 3) [47]. Environmental factors (slope, aspect, slope position, altitude, and GPS geographical coordinates) were also recorded for each sample plot with using a hand-held geological compass and a Garmin GPS receiver. About 1.0 kg of soil was collected from the corresponding soil layer at each location and returned to the laboratory to dry them under indoor natural ventilation condition. Samples were then ground and sieved them before chemical analyses. According to relative conferences [48–51], we could get the following knowledge: comprehensive evaluation of urban soil fertility is an effective means to judge urban soil quality. Soil fertility is the essential characteristics of soil, and a comprehensive reflection of soil physical, chemical and biological characteristics, among which soil pH, soil organic matter, nitrogen (N), phosphorus (P) and potassium (K) and their available state are very important soil properties for urban trees. Hence, we selected these eight soil chemical properties (SCPs), i.e., soil pH, soil organic matter (OM), total nitrogen (TN), total phosphorus (TP), total potassium (K), available nitrogen (AN), available phosphorus (AP), and available potassium (AK) as the soil fertility parameters. Details of the determination of the eight soil chemical properties are given in Table 2.

**Table 1:** Overview of the 95 sample plots used in this study.

No.of sample plots	Names of location	Latitude (N)	Longitude (E)	No.of sample plots	Names of location	Latitude (N)	Longitude (E)
1	Qidaoyaozigou	36°23′30″	109°38′37″	49	Yinchagou	36°17′53″	109°36′8″
2	Qidaoyaozigou	36°23′9″	109°39′51″	50	Yangpan	36°16′41″	109°34′41″
3	Qidaoyaozigou	36°22′12″	109°40′36″	51	Yangpan	36°15′7″	109°41′26″
4	Shenshan	36°20′15″	109°44′41″	52	Mengjiagou	36°17′40″	109°42′8″
5	Qidaoyaozigou	36°21′37″	109°41′58″	53	Zhangjiagoucun	36°16′28″	109°36′40″
6	Mafang	36°21′16″	109°43′6″	54	Chaiyacun	36°41′15″	109°37′12″
7	Danangou	36°19′11″	109°41′39″	55	Yansiwancun	36°44′43″	109°26′50″
8	Yangjiapan	36°28′37″	109°33′40″	56	Yushuwan	36°19′47″	109°37′6″
9	Wayaogou	36°21′25″	109°38′27″	57	Taobaoyu	36°19′38″	109°35′16″
10	Laowagou	36°24′39″	109°29′4″	58	Taobaoyu	36°19′40″	109°33′53″
11	Nanchuan	36°29′24″	109°26′30″	59	Jingzigou	36°21′12″	109°34′12″
12	Huaishuwacun	36°30′57″	109°26′27″	60	Taobaoyu	36°19′45″	109°32′48″
13	Shangjiagou	36°29′14″	109°28′20″	61	Nanpanlongcun	36°21′54″	109°32′5″
14	Beigoucun	36°32′21″	109°25′4″	62	Nanpanlongcun	36°22′14″	109°33′8″
15	Daluogou	36°27′50″	109°25′7″	63	Luogoucun	36°44′27″	109°39′54″
16	Lucaogou	36°25′50″	109°25′36″	64	Fenghuangshan	36°35′33″	109°28′32″
17	Wanhuashan	36°31′41″	109°21′2″	65	Qingliangshan	36°37′42″	109°27′52″
18	Jiuyanshan	36°26′18″	109°22′37″	66	Qingliangshan	36°36′37″	109°29′33″
19	Dongxinmaotaicun	36°28′37″	109°23′8″	67	Gaojiayuanze	36°34′19″	109°25′48″
20	Masichuangou	36°30′24″	109°37′52″	68	Suoyacun	36°33′22″	109°24′49″
21	Baitai	36°34′48″	109°39′57″	69	Xinyaocun	36°33′4″	109°26′44″
22	Pingtoushan	36°31′28″	109°34′58″	70	Majiawan	36°33′51″	109°27′36″
23	Xiyaogou	36°32′48″	109°36′27″	71	Xiejiagou	36°40′3″	109°26′38″
24	Songshulincun	36°33′7″	109°31′14″	72	Lijiawacun	36°37′43″	109°26′40″
25	Yingpanshan	36°34′36″	109°22′29″	73	Xiaobiangou	36°36′30″	109°26′57″
26	Peizhuang	36°37′1″	109°24′56″	74	Fenghuangshan	36°36′9″	109°27′57″
27	Dongyuzitan	36°40′46″	109°17′20″	75	Yangou	36°34′2″	109°29′35″
28	Liuqu	36°39′29″	109°19′49″	76	Miaoancun	36°33′32″	109°29′51″
29	Liuqiaogou	36°37′56″	109°22′31″	77	Yangou	36°32′55″	109°28′44″
30	Laofoyegeda	36°42′47″	109°31′44″	78	Baotashan	36°34′49″	109°29′3″
31	Wangjiabiancun	36°40′10″	109°32′0″	79	Yehuzigou	36°35′43″	109°30′5″
32	Qingliangshan	36°37′36″	109°29′50″	80	Yehuzigou	36°36′1″	109°30′53″
33	Zhaoyaocun	36°37′30″	109°34′11″	81	Qingliangshan	36°37′5″	109°29′0″
34	Luojiaping	36°23′21″	109°35′54″	82	Shangheniancun	36°32′55″	109°19′29″
35	Panlongshan	36°23′51″	109°32′50″	83	Zuocunyugou	36°31′56″	109°18′45″
36	Jutuangou	36°23′56″	109°34′45″	84	Baotashan	36°35′42″	109°29′33″
37	Yehuzigou	36°35′47″	109°31′58″	85	Baotashan	36°34′46″	109°29′29″
38	Liugou	36°28′50″	109°16′58″	86	Baotashan	36°35′12″	109°29′30″
39	Huoshigou	36°31′49″	109°15′24″	87	Gaojiayuanze	36°34′32″	109°28′19″
40	Houjiulongquan	36°12′15″	109°36′44″	88	Lingmaoshan	36°35′12″	109°26′57″
41	Houjiulongquan	36°12′15″	109°36′44″	89	Jiuzigou	36°26′11″	109°31′23″
42	Houjiulongquan	36°14′22″	109°37′16″	90	Yehuzigou	36°35′9″	109°31′9″
43	Houjiulongquan	36°12′41″	109°35′26″	91	Qingliangshan	36°36′17″	109°29′5″
44	Xiaonangou	36°15′1″	109°35′11″	92	Wanhuashan	36°32′12″	109°20′22″
45	Houjiulongquan	36°13′53″	109°35′33″	93	Wanhuashan	36°32′25″	109°20′39″
46	Shuiweigou	36°17′38″	109°44′8″	94	Wanhuashan	36°31′59″	109°20′29″
47	Makeyigou	36°39′45″	109°44′35″	95	Wanhuashan	36°32′9″	109°20′37″
48	Yangchagou	36°18′6″	109°34′36″				

**Table 2:** Methods used to analyze the soil chemical properties.

Soil chemical properties	Methods (references)	Analytical instruments (type, manufacturer)
Total nitrogen (TN)	Kjeldahl method (dissolved by sulfuric acid plus catalyst)	Fully automated Kjeldahl analyzer (FOSS-8400, Germany)
Total phosphorus (TP)	Dissolved using nitric acid, perchloric acid, and hydrofluoric acid [52]	Automatic discontinuous analyzer (Clever chem200, Germany)
Available nitrogen (AN)	0.01 N CaCl_2_ extraction [53]	Same as TP
Total potassium (TK)	Dissolved using nitric acid, perchloric acid, hydrofluoric acid [54,55]	Same as TP
Available phosphorus (AP)	0.5 M NaHCO_3_ extraction [56]	Ultraviolet spectral photometer (UV-1780, Shimadu, Tokyo, Japan)
Available potassium (AK)	1 N NH_4_OAC extraction [56]	Flame photometer (FP640, Shanghai, China)
Total organic matter (OM)	K_2_Cr_2_O_4_ volumetric method [57]	Oil bath-K_2_CrO_7_ titration method
pH	1:2.5 water-soluble extract [58]	pH meter (Mettler-Toledo, Switzerland)

### Sample plot vegetation survey

The canopy closure, stem height (height of the first major branch), tree height, diameter at breast height (DBH), crown width, plant community structure, and health status were surveyed for the trees in each plot in 95 sample plots (20 m × 20 m) when the soil samples were collected (S1 Table and S2 Table). These parameters were determined according to the Chinese Forestry Standards “Observation Methodology for Long-term Forest Ecosystem Research” (LY/T 1952–2011). Our field surveys and soil sampling procedures were authorized by local forestry administration (Forestry Bureau of Baota District in Yan’an City). Our study only involved soils and plants, and no humans or animals, and did not involve endangered or protected species.

### Semivariance model optimization and spatial interpolation

In this study, empirical semivariogram values were obtained for the eight soil chemical properties using Eq 1:

$$\gamma (h)=\frac{1}{2N(h)}\sum_{i=1}^{N(h)}{\left[Z(Xi)-Z(Xi+h)\right]}^{2},$$ (1)

where *γ*(*h*) is the sample semivariance between all observations *Z*(*Xi*), *Z* is the measured value at a particular location, *N*(*h*) represents the number of paired data at distance *h*, and (*h*) is the lag distance that separates the total numbers of data pairs. The semivariogram may be fitted with spherical, exponential, Gaussian, or linear models (Eqs 2–5, respectively):

$$\gamma (h)=\left\{\begin{array}{cc} C_{0}+C\left[\frac{3h}{2a}-\frac{h^{3}}{2a^{3}}\right], & h\leq a\\ C_{0}+C, & ha \end{array}\right\},$$ (2)

$$\gamma (h){=C}_{0}+C\left[1‑\exp\left(-\frac{h}{a}\right)\right],h\geq 0,$$ (3)

$$\gamma (h){=C}_{0}+C\left\{1‑\exp\left(-\frac{h^{2}}{a^{2}}\right)\right\},h\geq 0,$$ (4)

$$\gamma (h)=\left\{\begin{array}{cc} C_{0}+ch, & 0<h<a\\ C_{0}+C, & h>a \end{array}\right\},$$ (5)

where C_0_ is the nugget (N), C + C_0_ is the sill (S), and *a* is the correlation length. The detailed meanings of semivariogram parameters, including a, C_0_, C and h were explained in relevant literatures [59–61].

#### 2.5.1 Ordinary kriging (OK)

Predictions are usually obtained by calculating some weighted average of the observations and the interpolation procedure is as follows, Eq 6:

$$\hat{Z}(S_{0})=\sum_{i=1}^{n}\lambda_{i}•Z(S_{i}),$$ (6)

where $\hat{Z}(S_{0})$ is the predicted value of the target variable at an unvisited location *S*_*0*_ given its map coordinates, the sample data *Z*(*S*_*1*_), *Z*(*S*_*2*_),…, *Z*(*S*_*n*_), and their coordinates. The weights λ_*i*_ are selected in order to minimize the prediction error variance, thereby yielding weights that depend on the spatial autocorrelation structure of the variable.

#### Regression kriging (RK)

Predictions were obtained by modeling the relationships between the target and auxiliary environmental variables at the sample locations, and by applying these relationships to unvisited locations by using the known values of the auxiliary variables at these locations. In order to obtain spatial predictions of the soil chemical properties with regression kriging, the usual auxiliary environmental predictors employed comprised the land surface parameters, remote sensing images [13,18], and geological information [8], soil data [19], and land-use maps [18]. In this study, we used terrain factors as auxiliary environmental predictors, as described in detail in Table 2. The principles of mathematical principles are shown in Eq 7 [62,63]:

$$\hat{Z}(S_{0})=\sum_{k=1}^{p}{\hat{\beta }}_{k}•q_{k}(S_{0})+\sum_{i=1}^{n}\lambda_{i}•e(S_{i}),$$ (7)

where *q*_*k*_ are the estimated regression coefficients, *p* is the number of predictors or auxiliary variables, *q*_*k*_ are the estimated drift model coefficients, *q*_*k*_(*S*_*0*_) are the values of the auxiliary variables at the target location, ${\hat{\beta }}_{0}$ is the estimated intercept, *λ*_*i*_ are kriging weights determined by the spatial dependence structure of the residual, and *e*(*S*_*i*_) is the residual at location *S*_*i*_. The regression coefficients ${\hat{\beta }}_{k}$ were estimated from the sample using a fitting method. In this study, we used the stepwise ordinary least squares method to estimate ${\hat{\beta }}_{k}$ with collinearity diagnostics in SAS. $\sum_{k=1}^{p}{\hat{\beta }}_{k}•q_{k}(S_{0})$ is the fitted drift, and $\sum_{i=1}^{n}\lambda_{i}•e(S_{i})$ is the interpolated residual. The detailed computational process employed for making regression kriging predictions was described in previous studies [18,19].

### Auxiliary terrain variables

Topography controls the flow of water, solutes, and sediments, thereby affecting soil development and the formation of the typical patterns of spatially distributed soil properties [64]. We calculated the relative elevation (*H*_*r*_), slope (*β*), aspect (*A*), sunny slope (*cosA*), shady slope (*sinA*), terrain wetness index (*TWI*), stream power index (*SPI*), vertical curvature (*C*_*v*_), horizontal curvature (*C*_*h*_), range of change in the elevation (*QFD*), macroscopic information on the surface form (*M*), and relative position index (*RPI*) based on the digital elevation model at a spatial resolution of 5 m according to the appropriate computing methods in ArcGIS 10.0 and SimDTA-V1.0.3 [65–67]. Further details of the computation of the terrain factors are given in Table 3.

**Table 3:** Topographical factors and their descriptions.

Topographical factors	Formula	Descriptions	References.
*H*_*r*_	*H*_*r*_ = *H*_*max*_-*H*	*Hmax* = maximum height in the area and *H* = absolute elevation	[68]
*β*	$\beta =\arctan\sqrt{{\left(\frac{\delta_{z}}{\delta_{x}}\right)}^{2}+{\left(\frac{\delta_{z}}{\delta_{y}}\right)}^{2}}$	*δ*_*x*_, *δ*_*y*_, *δ*_*z*_ are the differences in distance at the *x*, *y*, and *z* orientations, respectively	[63]
*A*	$A=\arctan(\frac{f_{y}}{f_{x}})$	*f*_*y*_, *f*_*x*_ are the rates of change in elevation in the north–south and east–west directions, respectively	[63]
cos*A*	cos*A =* cos(Aspect), Aspect = *A*	Sunny slope	[69]
sin*A*	sin*A =* sin(Aspect), Aspect = *A*	Shady slope	
*C*_*v*_	*C*_*v*_ = Slope of slope (SOS)	Slope = *β*, and *C*_*v*_ is a proxy for the second derivative of the change in ground elevation.	[70]
*C*_*h*_	*C*_*h*_ = Slope of aspect (SOA)	*C*_*h*_ is a proxy for bending and variations in the surface of the earth in horizontal directions	[70]
*QFD*	*QFD* = Maxn—Min_n_	Range of change in the surface elevation	[48]
*M*	*M* = cos^−1^(*β*×3.1415/180)	Terrain roughness on the surface	[48]
*RPI*	$RPI=\frac{ED_{nv}}{ED_{nv}+ED_{nr}}$	*ED*_*nv*_, *ED*_*nr*_ are proxies for the Euclidean distance to the nearest valley and to the nearest ridge, respectively	[66]
*TWI*	*TWI* = ln(*A*_*s*_/tan *β*)	*A*_*s*_ is the cumulative upslope area per unit contour length (or specific catchment area) and β is the slope gradient in radians	[71,72]
*SPI*	*SPI* = *A*_*s*_×tan *β*	*As* and *β* are the same as above	

### Model validation

We performed leave-one-out cross-validation [3,24–26,28]. This process was repeated for all of the observations. Three standard indices comprising the mean error (ME), mean relative error (MRE), and root mean squared error (RMSE) were used to compare the accuracy of interpolation. These indices were calculated as follows with Eqs 8, 9 and 10 [3,24,25,73]:

$$\mathrm{ME}=\frac{1}{m}\sum_{j=k}^{m}\left(Z_{k}‑{\hat{Z}}_{k}\right),$$ (8)

$$\mathrm{MRE}=\frac{1}{m}\sum_{k=1}^{m}\frac{\left|Z_{k}‑{\hat{Z}}_{k}\right|}{Z_{k}}\times 100,$$ (9)

$$\mathrm{RMSE}=\sqrt{\frac{1}{m}\sum_{k=1}^{m}{\left(Z_{k}‑{\hat{Z}}_{k}\right)}^{2}},$$ (10)

where *m* is the number of points, Z_*k*_ is the observed content of the *k*th measurement, and ${\hat{Z}}_{k}$ is the predicted value of the *k*th measurement. ME is a measure of the bias of the interpolation, which should be close to zero for unbiased methods, whereas MRE and RMSE are measures of prediction accuracy, which should be as low as possible.

### Soil fertility assessment

#### The assessment of the enrichment and the paucity for every single soil chemical property

The time-consuming collection of detailed objective soil content measures is justified when biophysical analysis is warranted. The classification criteria for the enrichment and the paucity of the eight soil chemical properties were assigned according to the second soil survey in Shaanxi province [48], as shown in Table 4.

**Table 4:** Classification criteria for the enrichment and the paucity of the eight soil chemical properties.

Grades	1	2	3	4	5	6	7	8
Comment	very high	higher	high	above average	below average	low	lower	very low
OM (g/kg)	>40	30–40	20–30	15–20	12–15	10–12	8–10	6–8
TN (g/kg)	>2.0	1.50–2.0	1.50–1.25	1.0–1.25	0.75–1.0	0.5–0.75	<0.5	-
TP (g/kg)	>1	0.80–1.0	0.6–0.8	0.4–0.6	0.2–0.4	<0.2	-	-
TK (g/kg)	>25	20–25	15–20	10–15	5–10	<5	-	-
AN (mg/kg)	>150	120–150	90–120	60–90	45–60	30–45	20–30	<20
AP (mg/kg)	>40	30–40	20–30	15–20	10–15	5–10	3–5	<3
AK (mg/kg)	>200	150–200	120–150	100–120	70–100	50–70	30–50	<30
pH (boreal)	>8.3	8.0–8.3	7.5–8.0	7.0–7.5	6.5–7.0	6.0–6.5	5.7–6.0	<5.7

#### Comprehensive soil fertility assessment based on the Nemerow index

In order to eliminate the dimensional differences among the soil properties, the eight soil chemical properties, comprising, AN, AP, AK, TN, TP, TK, OM, and pH, were tested and nondimensionalized according to the following computational formulae, Eq 11:

$$\{\begin{array}{ccc} F_{i}=c_{i}/x_{a}, & c_{i}\leq x_{a}, & \left(F_{i}\leq 1\right)\\ F_{i}=1+(c_{i}-x_{a})/(x_{c}-x_{a}), & x_{a}<c_{i}\leq x_{c} & \left(1<F_{i}\leq 2\right)\\ F_{i}=2+(c_{i}-x_{c})/(x_{p}-x_{c}), & x_{c}<c_{i}\leq x_{p}, & \left(2<F_{i}\leq 3\right)\\ F_{i}=3 & c_{i}>x_{p} & \end{array},$$ (11)

where *F*_*i*_ is the nondimensionalized fertility index of *i* soil properties, *C*_*i*_ are the measured values of *i* soil properties estimated by ordinary kriging or regression kriging at a spatial resolution of 5 m, and *x*_*a*_, *x*_*c*_, and *x*_*p*_ are the indices for the eight soil chemical properties, as shown in Table 5.

**Table 5:** Grading criteria for eight soil chemical properties using the Nemerow index grading method.

	Soil nutrients and chemical properties							
Classification index of Nemerow	TN	TP	TK	AN	AP	AK	OM	pH (>7.0)
***x***_***a***_	0.75	0.4	5	60	5	50	10	9
***x***_***c***_	1.5	0.6	20	120	10	100	20	8
***x***_***p***_	2	1	25	180	20	200	30	7

The comprehensive soil fertility index (F), was calculated as follows:

$$F=\sqrt{\frac{{\left({\bar{F}}_{i}\right)}^{2}+{\left(F_{i\min}\right)}^{2}}{2}}•(\frac{n-1}{n}),$$ (12)

where ${\bar{F}}_{i}$ is the average value of each fertility index, *F*_*i*min_is the minimum value of each fertility index, and *n* is the index number [49,50]. i.e., *n* = 8 in this study. Soil fertility grading was based on the method of Kan and Wu (1994), as shown in Table 6 [50]. We improved Nemerow index (F) and replaced the maximum value for a single factor in the original Nemerow index (F0), which is mainly used for soil pollution assessment, with the minimum value of single factor based on the Liebig's law of the minimum. In the improved Nemerow index, additional amendments (n –1)/n are used to increase the credibility of the evaluation.

**Table 6:** Grading criteria for various soil properties in the Nemerow grading method.

Soil fertility grade	Class I (very fertile)	Class II (fertile)	Class III (general)	Class IV (barren)
F	F ≥ 2.70	1.80 ≤ F < 2.70	0.90 ≤ F < 1.80	F < 0.90

### Softwares used for statistical analysis and mapping

The Kolmogorov–Smirnov normal distribution test and statistical analyses were perfoemed with in the SAS 9.2. Semivariogram analysis was conducted using GS+ 7.0. Spatial interpolation and mapping were performed with ArcGIS 10.0.

## Results

### General normality analysis and optimization of semivariograms parameters for soil chemical properties

Table 7 shows the descriptive statistics (maximum, minimum, mean, standard deviation, coefficient of variation, skewness and kurtosis) obtained from the modeling dataset for the eight soil chemical properties at three soil depths. In general, the average value of each of the soil chemical properties decreased as the soil depth increased in the 0–60 cm soil profile with 20 cm intervals (Table 7). The skewness is a statistical index that indicates the degree of the symmetrical distribution for data. The skewness of a normal distribution is zero, and the tail length is symmetrical on both sides. If the skewness is positive, the data are relatively scattered on the right side of the mean. Otherwise, the data on the left side of the mean are more diffuse [74]. The raw data of TN (40–60 cm), TP (40–60 cm), TK (40–60 cm), and pH were less than one, so these data all conformed to normal distributions and they could be used to directly the optimize relevant parameters (nugget, partial sill, sill, range, and nugget/sill) for the semi-variance functions. By contrast, a natural logarithmic transformation should be considered where the data’s skewness is larger than 1.0 [3]. Logarithmic transformations were performed for TN (0–20 cm, 20–40 cm), TP (0–20 cm, 20–40 cm), TK (0–20 cm, 20–40 cm), AN, AP, AK, and OM to make the data suitable for optimizing the semi-variance function model. The majority of measured soil chemical properties exhibited moderate spatial dependency according to the N/S value in semi-variance function models, where specific parameters used in this study are shown in S3 Table, S1 and S2 Figs.

**Table 7:** Descriptive statistics for soil nutrients and chemical parameters in the study area.

Data sets	SCPs	depth (cm)	Max	Min	Mean	SD	CV (%)	Raw data			Log-transfo rmed data	
								S.		K.	S.	K.
**Modeling data set** **(*n* = 70)**	TN	0–20	2.28	0.31	0.99	0.49	49.55	1.05		–0.72	–0.13	–1.26
	TN	20–40	1.36	0.01	0.55	0.26	47.69	1.21		1.61	0.57	–0.23
	TN	40–60	1.29	0.01	0.43	0.21	49.66	1.00		3.41	–3.48	18.62
	TP	0–20	1.05	0.34	0.71	0.18	24.78	1.17		–0.58	–0.86	–0.02
	TP	20–40	1.00	0.29	0.70	0.18	25.57	1.33		–0.46	–1.14	0.57
	TP	40–60	0.97	0.29	0.69	0.17	24.96	-0.61		–0.46	–1.11	0.51
	TK	0–20	16.86	9.60	11.90	1.95	16.36	1.11		–0.15	0.97	–0.41
	TK	20–40	24.86	14.44	20.19	2.84	14.07	1.30		–0.38	–1.06	–0.20
	TK	40–60	16.36	9.46	11.75	2.04	17.40	0.94		-0.20	1.01	–0.41
	AN	0–20	120.10	4.01	28.74	24.14	84.00	1.88		3.66	0.24	–0.35
	AN	20–40	73.80	3.66	17.34	15.67	90.34	2.21		4.87	0.64	–0.03
	AN	40–60	44.30	1.87	11.92	8.25	69.22	2.04		4.43	0.41	0.88
	AP	0–20	7.54	0.68	3.27	1.61	49.34	1.25		0.32	-0.36	0.07
	AP	20–40	6.41	0.04	2.45	1.44	58.51	1.31		0.37	-2.21	10.00
	AP	40–60	8.38	0.43	2.32	1.59	68.58	1.71		3.86	-0.14	–0.28
	AK	0–20	237.47	43.50	119.42	45.34	37.96	1.10		-0.32	-0.29	–0.50
	AK	20–40	212.54	15.80	82.24	40.12	48.79	1.06		0.76	-0.15	0.57
	AK	40–60	167.30	20.40	68.51	30.33	44.27	1.60		2.79	0.18	1.00
	OM	0–20	42.28	3.55	14.51	8.64	59.55	1.08		0.28	-0.07	–0.93
	OM	20–40	23.25	1.70	8.71	5.10	58.57	1.07		0.79	-0.32	–0.23
	OM	40–60	23.83	1.30	6.74	3.95	58.58	1.55		4.94	-0.54	0.03
	pH	0–20	8.39	7.21	7.68	0.28	3.60	0.64		0.12	0.55	–0.01
	pH	20–40	8.49	7.18	7.86	0.27	3.47	0.53		0.65	0.41	0.65
	pH	40–60	8.49	7.64	7.97	0.22	2.70	0.92		0.65	0.96	0.56

SCPs, soil chemical properties; Max, maximum; Min, minimum; SD, standard deviation; CV, coefficient of variation, S, skewness; K, kurtosis.

### Effects of terrain factors on eight soil chemical properties in three soil layers

The multiple linear stepwise regression (MLSR) models for different soil chemical properties are presented in Table 8. All of the regression models were significant (P < 0.05). The predicted residual sum of squares (PRESS) and the determination coefficients (R^2^) of MLR models ranged from 0.03 to 37.22 and from 0.32 to 0.90, respectively. The terrain variables in bold in Table 8, such as, ***RPI***, ***TWI***, ***sinA***, ***cosA***, ***M***, ***QFD***, and ***TWI*** had great impact on the soil chemical properties in the corresponding MLR models. *RPI* had significant negative correlations with TN in the 0–40 cm soil layer, with AP in the 0–20 cm soil layer, and with OM in the 0–60 cm soil layer, where these correlations became weaker as the soil depth increased. ***sinA*** had significant negative correlations with TN in the 0–20 cm soil layer, and AN in the 0–40 cm soil layer. ***M*** had significant positive correlations with AN and AP in the 20–60 cm soil layer, and with AK in the 0–20 cm soil layer, but a significant negative correlation with OM in the 0–20 cm soil layer. ***QFD*** had significant positive correlations with AP and AK in the 40–60 cm soil layer, and with OM in the 0–20 cm soli layer. ***TWI*** had a significant positive correlation with TN in the 20–40 cm soil layer, but a significant negative correlation with OM in the 0–20 cm soil layer.

**Table 8:** Multiple linear regression results base on the soil chemical properties and terrain factors.

Soil chemical properties	Soil layer depth (cm)	Multiple linear regression model	PRESS	R^2^
TN	0–20	**1.18715**–0.48469 × ***RPI****–* 0.13183 × *sinA*	12.0205	0.6337
TN	20–40	**0.62790**–0.17967 × ***RPI***	4.6838	0.3215
TN	40–60	0.03945 × ***TWI*** + 0.00099684 × *Hr*	2.3317	0.7280
TP	0–20	**0.55701** + 0.00138 × *SOA* + 0.00386 × *β*	1.7004	0.5049
TP	20–40	**0.53051** + 0.00163 × *SOA* + 0.00432 × β	1.8824	0.8433
TP	40–60	**0.52451** + 0.00142 × *SOA* +0.00415 × *β*	1.7918	0.8560
TK	0–20	**13.26704**–0.00572 × *Hr*	36.66964	0.9046
TK	20–40	**19.67484**–0.00495 × *A* + 0.05583 × *β*	27.1782	0.8824
TK	40–60	**13.31315**–0.00657 × *Hr*	28.1865	0.8877
AN	0–20	**27.83289**–6.55654 × ***sinA***	34.6380	0.3950
AN	20–40	–0.04631 × *A*–9.07759 × ***sinA*** + 45.25216 × ***M*–** 0.05992 × *Hr* –0.36048 × *β*	12.5840	0.6612
AN	40–60	**5.95783**–3.05316 × ***cosA*** +23.27171 × ***M***– 0.04351 × *Hr*–0.27508 × *β*	31.9609	0.7660
AP	0–20	**3.00305** + 0.00332 × *Hr* –1.10442 × ***RPI***	1.5488	0.4923
AP	20–40	**1.73997**–0.00341 × *A* + 3.39581 × ***M*–** 0.00526 × *Hr*– 0.03875 × *β*	0.9877	0.6330
AP	40–60	**1.76704** + 4.48756 × ***M***– 0.00662 × *Hr* + 0.50955 × ***QFD***– 0.20137 × *β* –0.01850 × *SOA*	1.1512	0.4245
AK	0–20	**84.10390**–0.10666 × *A* + 51.44872 × ***M***	1.1439	0.3294
AK	20–40	**69.25850**–0.09693 × *A* + 0.11056 × *Hr*	0.8503	0.3786
AK	40–60	**59.49727**–0.11319 × *A* + 0.07911 × *Hr* + 2.30561 × ***QFD***– 0.27430 × *SOS*	0.4789	0.5798
OM	0–20	**11.85093** + 3.69587 × *cosA*– 12.24428 × ***M*** + 0.06302 × *Hr* + 0.53859 × *QFD*– 5.77321 × ***RPI*** + 0.12594 × *SPI*– 1.20692 × ***TWI***	37.2217	0.7160
OM	20–40	**7.28906**–0.01283 × *A* + 0.02654 × *Hr*– 3.79607 × ***RPI***– 0.06848 × *SOS*	12.2581	0.7208
OM	40–60	**6.01562**–0.01078 × *A* + 0.01810 × *Hr*– 2.19660 × ***RPI***– 0.05297 × *SOS*	7.5727	0.6993
pH	0–20	**7.56681** + 0.00065837 × *A*	0.0413	0.7666
pH	20–40	**7.78612** + 0.00044026 × *A*	0.0415	0.6042
pH	40–60	**7.87799** + 0.00051648 × *A*	0.02799	0.7832

PRESS, predicted residual sum of squares; R^2^, coefficient of determination; the intercept and estimated parameters in the regression models were all significant at *P* < 0.05, in two-tailed tests. The terrain variables and the intercept in bold significantly affected the corresponding dependent variable (soil chemical properties) in the multiple linear stepwise regression models because their absolute regression coefficient were larger than 0.1.

### Comparison of two spatial interpolation methods for eight soil chemical properties at the urban forest scale

#### Validation of predictive accuracy using ordinary kriging and ordinary kriging for the eight soil chemical properties

The predictions accuracy are shown in Table 9. The variogram for the soil chemical properties had the lowest nugget (*C*_*0*_) variance for TN (0–20 cm depth), followed by TN (40–60 cm depth) and AK (0–20 cm depth) using the OK method. The nugget value (1.872) for TK in the 40–60 cm soil layer was the largest compared with the other soil chemical properties, thereby demonstrating the great variations in this value over small distances. The small nugget value for almost all of the soil chemical properties indicated that the sampling density was adequate for determining the spatial structure, e.g., TN in the soil layers of 0–20 cm and 40–60 cm soil layers, and AK in the 0–20 cm soil layer. The range of the soil chemical properties in the three soil layers varied with the distance from 1290 m to 71100 m (S1 and S2 Figs). The predictive accuracies of ordinary kriging and regression kriging performed were good based on the lower ME, MRE, and RMSE. Our results showed that regression kriging performed better than ordinary kriging for TN (0–20 cm and 40–60 cm), OM (0–20 cm and 20–40 cm), and AP (20–40 cm and 40–60 cm). By contrast, ordinary kriging performed better than regression kriging for AN, AK, TP, TK, and pH of in three soil layers, and TN, OM, and AP of in other soil layers (Table 9).

**Table 9:** Accuracy of the spatial predictions of the eight soil chemical properties in three soil layers between ordinary kriging and regression kriging.

Soil chemical properties	Soil depth (cm)	ordinary kriging			regression kriging			Preferred kriging method ^a)^
		ME	MRE	RMSE	ME	MRE	RMSE	
TN	0–20	0.1745	0.3051	0.5011	0.1017	0.2726	0.4941	regression kriging
TN	20–40	–0.0394	0.2530	0.1712	0.0618	0.2952	0.1898	ordinary kriging
TN	40–60	–0.0010	0.4567	0.2109	0.0418	0.6501	0.2961	regression kriging
TP	0–20	0.0319	0.1842	0.1459	–0.0612	0.4297	0.2863	ordinary kriging
TP	20–40	–0.0023	0.2307	0.1558	–0.0405	0.2985	0.1820	ordinary kriging
TP	40–60	–0.0077	0.2075	0.1302	–0.0287	0.2409	0.1488	ordinary kriging
TK	0–20	0.4253	0.0633	1.0103	0.6928	0.1137	1.9418	ordinary kriging
TK	20–40	–0.3428	0.0627	1.5210	–0.5747	0.0822	1.8376	ordinary kriging
TK	40–60	0.2707	0.0658	1.0864	0.6032	0.0882	1.3630	ordinary kriging
OM	0–20	–0.0245	0.3429	5.3664	0.0199	0.3133	4.9037	regression kriging
OM	20–40	–0.1601	0.3390	2.9028	–1.7600	0.5545	4.9456	regression kriging
OM	40–60	0.4096	0.3528	2.3882	–0.7419	0.5245	2.9895	ordinary kriging
AN	0–20	2.9597	0.3976	16.8738	7.2789	0.8229	24.0490	ordinary kriging
AN	20–40	–1.6194	0.572	9.0432	–5.1876	0.9160	10.3496	ordinary kriging
AN	40–60	1.3670	0.4171	6.8469	–1.7140	0.6293	7.8552	ordinary kriging
AP	0–20	0.4377	0.5334	2.3738	–0.1243	0.6465	2.3925	ordinary kriging
AP	20–40	0.5608	0.7391	1.9435	–0.4369	0.5900	1.2652	regression kriging
AP	40–60	0.1486	1.5290	1.4298	–0.1317	1.5209	1.3270	regression kriging
AK	0–20	–5.4295	0.4672	38.1614	–19.9949	0.6139	46.5479	ordinary kriging
AK	20–40	–11.9706	0.5414	26.9388	–23.1926	0.7430	34.1939	ordinary kriging
AK	40–60	–13.3555	0.5231	20.8804	–22.4035	0.7115	29.2811	ordinary kriging
pH	0–20	0.0182	0.0192	0.2419	0.0269	0.0203	0.2384	ordinary kriging
pH	20–40	–0.0001	0.0225	0.2329	0.0141	0.0219	0.2260	ordinary kriging
pH	40–60	–0.0024	0.0186	0.2016	0.0015	0.0186	0.2020	ordinary kriging

^a)^ Summarized based on Table 7 and Fig 4.

#### Comparison of spatial distribution mapping for the eight soil chemical properties using ordinary kriging and regression kriging

**Fig 4:**
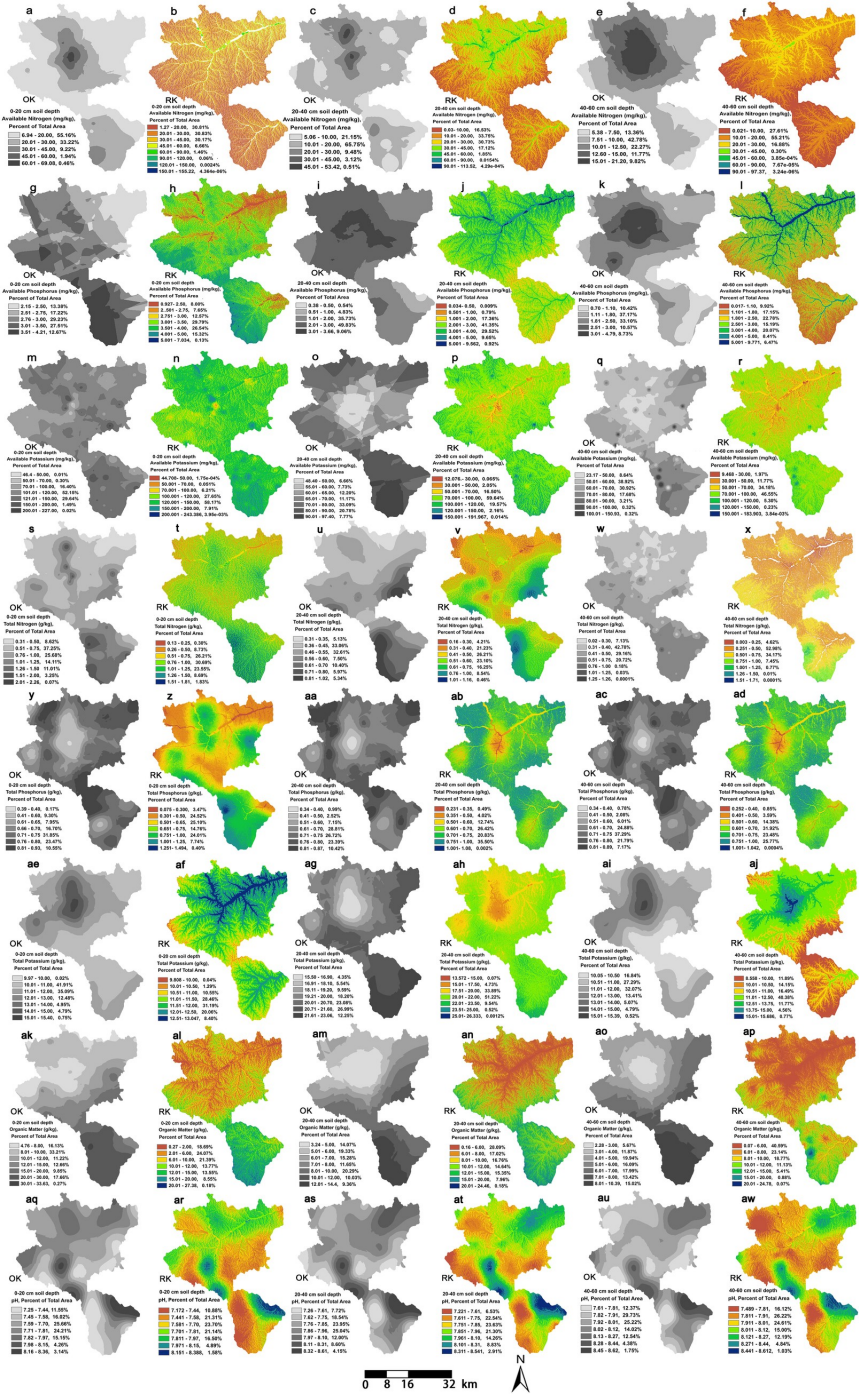
Spatial predictions of the eight soil chemical properties of different soil layer depth obtained by ordinary kriging (gray maps) and regression kriging (colored maps). **(**a–f), available nitrogen; (g–l), available phosphorus; (m–r), available potassium; (s–x), total nitrogen; (y, z, aa–ad), total phosphorus; (ae–aj), total potassiumTK; (ak–ap), organic matter; and (aq–aw), pH.

The spatial mapping technique was employed to assess the extent and magnitude of spatial heterogeneity of the soil chemical properties and fertility, especially soil nutrients and their deficiencies. Fig 4 shows that the general spatial distributions of the eight soil chemical properties determined by the two kriging interpolation methods were similar in the three soil layers. However, there were some differences between the two predictions. Firstly, the ranges of the predictions obtained by regression kriging were larger than those using ordinary kriging. Second, the vector polygons were more fragmented in the regression kriging maps, which were more sensitive to the variations in terrain and vegetation. The best kriging interpolation method for specific soil chemical properties are indicated in Table 9 (ninth column), mainly based on the topographical features, vegetation cover types, prediction accuracy (Table 7), and the continuity of the spatial distribution maps (Fig 4).

Fig 4A, Fig 4B and 4E indicates that AN contents were generally low in the three soil layers, and AN was highly deficient at the margin of the study area with steep terrain. Fig 4G, Fig 4J and 4L indicates that the AP distributions were similar to these of AN. However, the AP values in the valley landform, especially, near Yanhe River, were higher than those in the other areas, mainly the river carried sediments containing phosphorus. AK was mainly above average in the surface and near-surface layers (Fig 4M and 4O). The AK contents of the subsurface layer were mainly low, and below average (Fig 4Q). In general, AK was highly deficient in the near-surface and subsurface layer soils in the low vegetation cover areas along the Yanhe River. Fig 4T indicates that the TN contents of the surface layers were generally low (accounting for 26.21% of the study area), below average (accounting for 30.69%), or above average (accounting for 23.55%). The TN contents of the near-surface layers were mainly low (accounting for 66.21%) or very low (accounting for 25.31%) (Fig 4U). The TN contents of the subsurface layers were mainly very low (accounting for 57.60%) or low (accounting for 34.17%). Spatially, the lower TN contents were usually distributed in farmlands before the implementation of the “Grain for Green” ecological restoration project in China. Fig 4Y, Fig 4Z, Fig 4Aa, Fig 4Ab, Fig 4Ac and 4Ad indicated that the TP contents of the surface layer, near-surface layer, and subsurface layer with predication by ordinary kriging were generally in the ranges of 0.39–0.93 g/kg, 0.34–0.87 g/kg, and 0.34–0.89 g/kg, respectively, where there were four grades comprised below average, above average, high, and very high,. In the whole study area, the soil TP contents in the 0–60 cm depth layer were sufficient for the existing agriculture and forestry requirements. Fig 4Ae, Fig 4Af, Fig 4Ag, Fig 4Ah, Fig 4Ai and 4Aj showed that the TK contents of the surface layer, near-surface layer, and subsurface layer were mostly in the ranges of 15.01–25.00 g/kg, 15.50–23.06 g/kg, and 10.05–15.39 g/kg, respectively, where most were above average. In the whole study area, the TK contents in the 0–60 cm depth layer were sufficient for the existing agriculture and forestry requirements. Fig 4Al, Fig 4Am, Fig 4An, Fig 4Ao and 4Ap shows that the OM range and contents decreased as the soil depth increased. In the whole study area, the OM contents in the 0–60 cm depth layer were insufficient for the existing agriculture and forestry requirements. Fig 4Ar, Fig 4As and 4At showed that the pH values in the surface layer and near-surface layer were mostly in the range of 7.00–8.00, where the two grades were above average (accounting for 93.53% and 84.18%, respectively). The pH in the subsurface layer was usually high in the range of 7.00–8.30, where the three grades comprised above average, high, and very high, and these three levels accounting 98.97% in total (Fig 4Aw). In the whole study area, the pH in the 0–60 cm depth soil layer was rather alkaline for existing agriculture and forestry utilization.

### Effects of vegetation cover types and soil layer depth on the eight soil chemical properties and the comprehensive fertility

**Fig 5:**
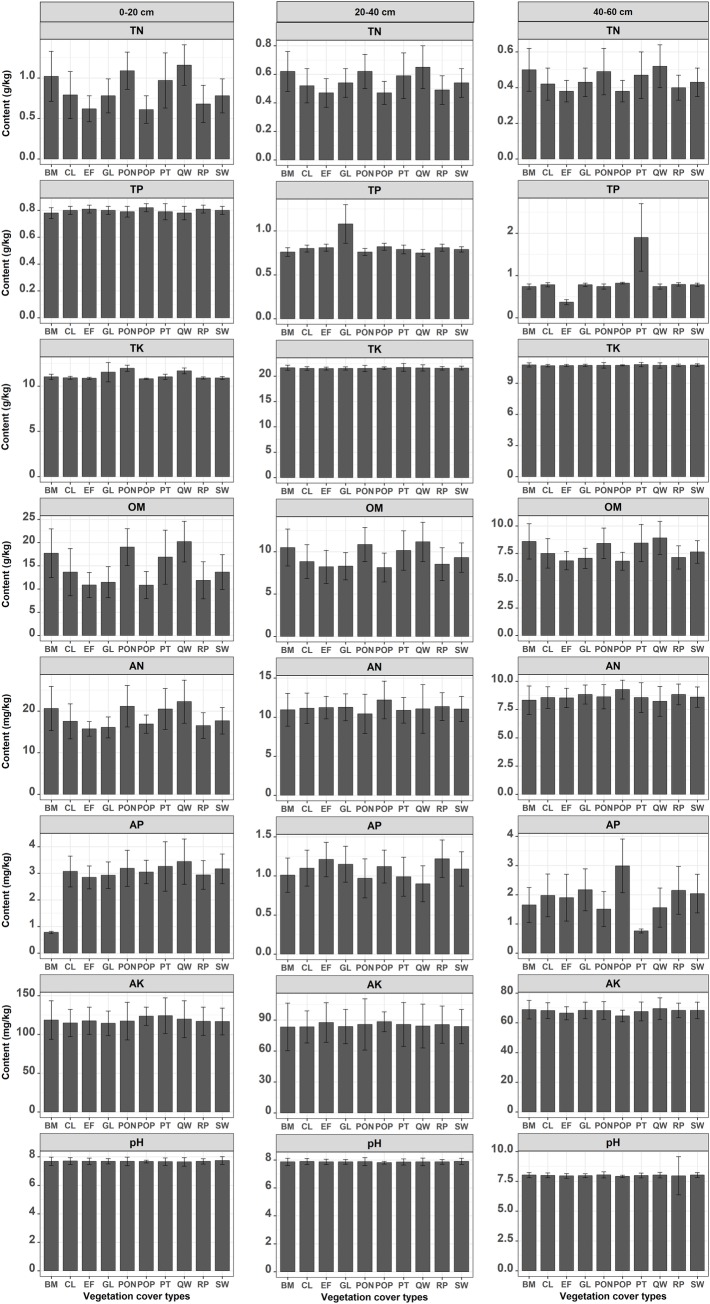
Average levels of eight soil chemical properties in the three soil layers in Yan’an urban forest under 10 vegetation cover types. BM, Broadleaved mixed natural forest; CL, Cultivated land; EF, Economic forest; GL, Grassland; PON, *Platycladus orientalis* natural forest; POP, *Platycladus orientalis* plantation forest; PT, *Pinus tabuliformis* plantation forest; QW, *Quercus wutaishanica* natural forest; RP, *Robinia pseudoacacia* plantation forest; SW, Shrubwood.

The effects of the vegetation cover types and soil layer depth on the eight soil chemical properties and the comprehensive fertility are shown in Fig 5. First, the TN, OM, AN, AP and AK differed significantly in the 10 vegetation cover types across the three soil layers. In particular, sites with *Quercus wutaishanica* natural forest (QW), *Platycladus orientalis* natural forest (PON), and broadleaved mixed natural forest (BM) had higher TN, OM, AN, and AK contents than the other vegetation cover types. By contrast, there were small differences in TK, AK, and pH among the ten vegetation cover types. Second, the soil layer depth had different effects on the eight soil chemical properties. The contents of TN, OM, AK and pH contents with the corresponding vegetation cover types decreased as the soil depth increased. However, TK was larger in near-surface layer than the other two soil layers.

### Soil comprehensive fertility

**Fig 6:**
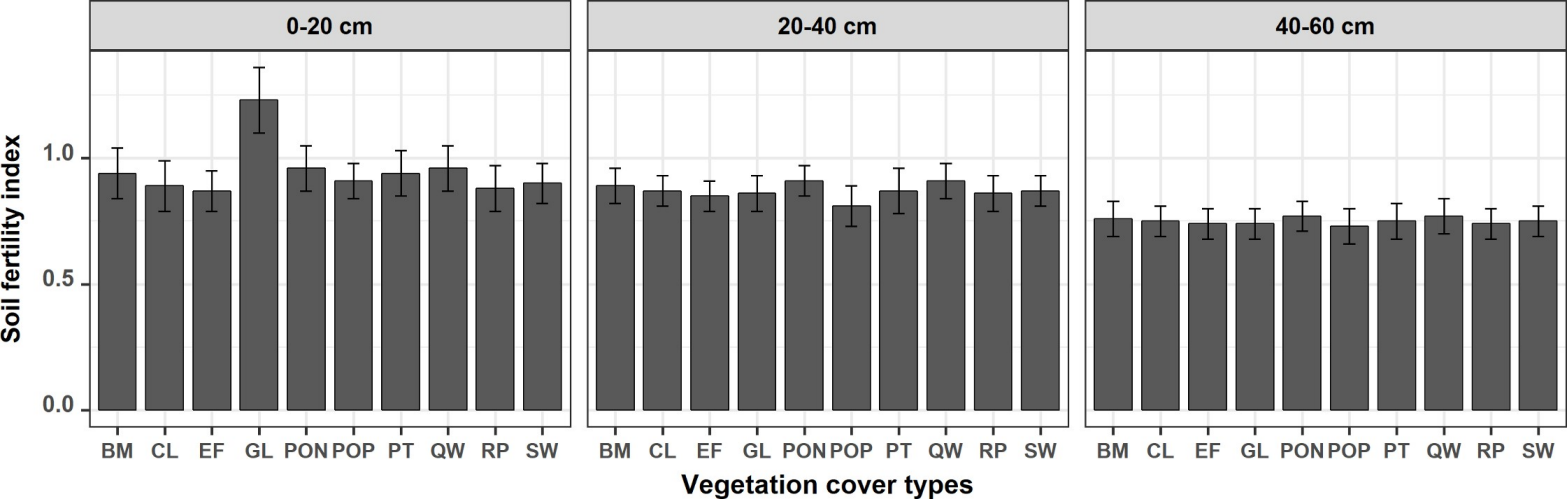
Average value of soil fertility index integrated the eight soil chemical properties (TN, TP, TK, AN, AP, AK, OM and pH) in Yan’an urban forest under different vegetation cover types at the three soil depth (0–20 cm, 20–40 cm, and 40–60 cm). The abbreviations for the vegetation cover types were the same as those used in the Fig 5.

The Fig 6 shows that the soil fertility index was the highest in the surface soil layer (0–20 cm) with the grassland (GL) because the soil chemical properties in most of the grassland areas were at the equilibrium state for natural cycling as artificial harvesting was performed and grazing was prohibited. The average F values for natural forests (PON, QW, BM, *Platycladus orientalis* plantation (POP), and shrubwood (SW)) were larger than 0.90, thereby indicating that the fertility at general level because of low human intervention in these ecosystems. The average F values for artificial vegetation types (cultivated land (CL), *Robinia pseudoacacia* plantation (RP), and economic forest (EF)) were less than 0.90, and thus their fertility was at the barren level due to major artificial harvesting from the CL and EF ecosystems. In addition, a dried soil layer was widespread in the RP forests due to their vigorous taproot systems and active transpiration, which reduced the plant diversity and slowed down the rate of litter decomposition on the topsoil. For the soil fertility index in near-surface soil layer (20–40 cm), the average F values for PON and QW were both 0.91 and at the general level because that PON and QW had both been near-natural forest for over 50 years so there was rich litters from the complex plant communities and their soil environmental conditions were suitable for the litter decomposition, while the soil void structure was better in the 0–40 cm soil layer than that under the other vegetation cover types. There were not obvious differences in the average F values among the other eight vegetation cover types because of their similar soil physical properties [75] The average F values for the subsurface soil layer (40–60 cm) were at the barren level, which may be attributed to the soil physical properties gradually homogenizing as the soil layer depth increased [76].

**Fig 7:**
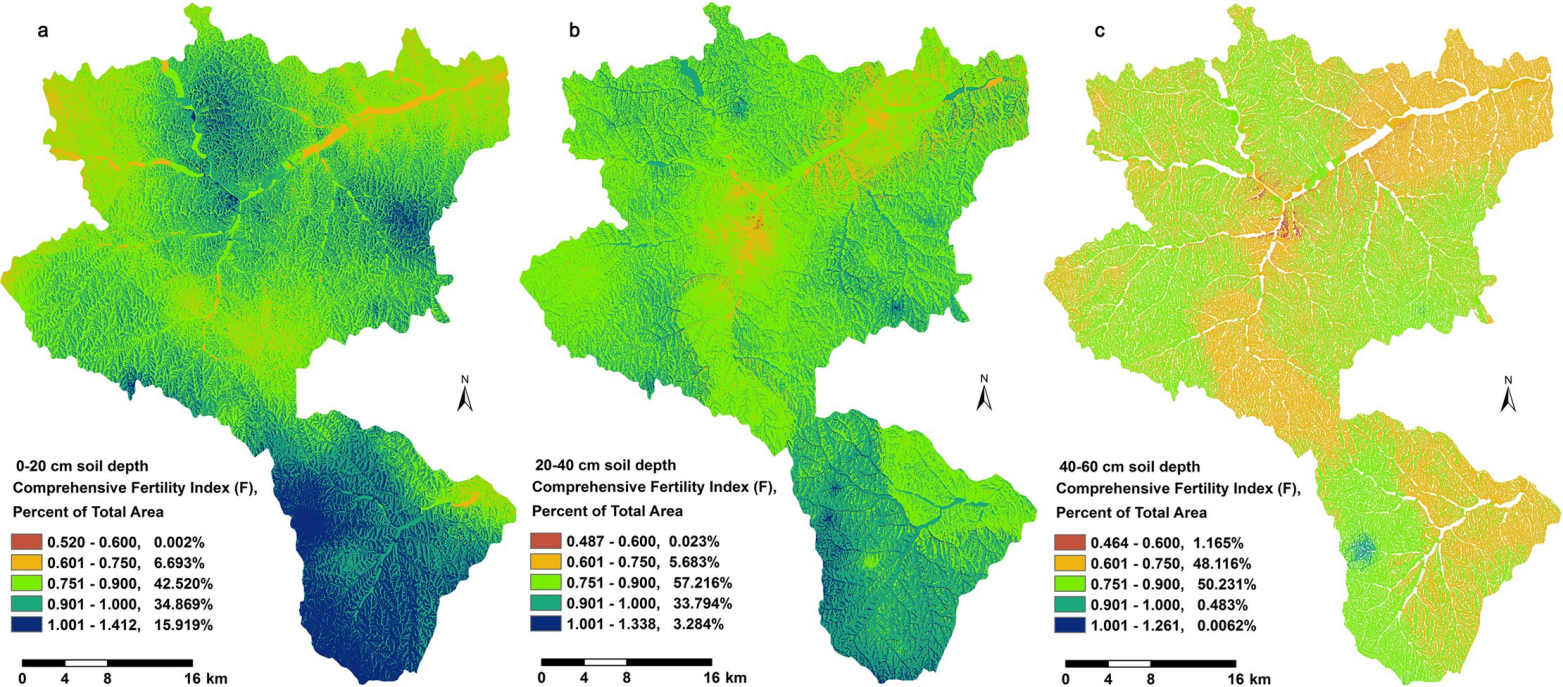
Spatial distribution maps for the soil comprehensive fertility index (F) integrated based on eight soil chemical properties in the three soil layers predicted using the high accuracy spatial interpolation method: (a) 0–20 cm soil layer, (b) 20–40 cm soil layer, and (c) 40–60 cm soil layer.

According to the selection results obtained by the kriging interpolation method (Table 9), the F values for the three soil layers were computed with ArcGIS 10.0, as shown in Fig 1B, Fig 7, Table 6, and Table 8. (1) Spatially, west of Nanniwan, east of Chuankou, and parts of Wanhuashan, Hezhuangping, and Yaodian were at the general level (0.90 ≤ F < 1.80) based on the perspective of the comprehensive fertility index (F). (2) In the vertical soil profile, the cover area percentage with the barren fertility level (F ≤ 0.90) tended to increase from the surface layer (0–20 cm depth) to the subsurface layer (40–60 cm depth). However, the cover area percentage with the general fertility level (0.90 ≤ F < 1.80) exhibited a declining trend. (3) The spatial distribution maps of soil comprehensive fertility (F index) clearly matched with the terrain texture, which may facilitate the efficiency of land utilization.

## Discussion

### Effects of vegetation cover types, terrain, and soil layer depth on the soil chemical properties

In this study, we found that the mixed forests (*Q*. *wutaishanica* + *P*. *orientalis*, *P*. *hopeiensis* + *R*. *pseudoacacia*, *R*. *pseudoacacia* + *P*. *davidiana*, etc.) had higher levels of AN, AP, AK, TN, TP, TK, and OM, compared with the pure plantations (S1 Table, and Fig 5). The average concentrations level of the single and comprehensive soil chemical properties in the forest stands with the three dominant tree species (*R*. *pseudoacacia*, *Q*. *wutaishanica* and *P*. *orientalis*) in S2 Table were higher than those with other tree species because of differences in the total number of plant species (TNPS) and DBH in S1 Table. Analysis using Pearson’s correlation coefficients based on the relationships between the soil chemical properties with TNPS and DBH (S4 and S5 Tables), showed that the TP contents in the 0–60 cm soil layer and TK in the 20–40 cm were negatively correlated with TNPS, whereas the TK and AN contents in 0–20 cm soil layer, and AN in the 40–60 cm were positively correlated with TNSP. In addition, the DBH was positively correlated with the concentration of TN in the 0–20 cm soil layer, AN and AK in all three soil layers, and OM and pH in the 0–40 cm soil layer. High vegetation cover and diversity (i.e., TNPS) are helpful for soil conservation [77]. Moreover, forest land can improve the availabilities of soil N and organic C, and *R*. *pseudoacacia* cou1d enhance the concentration of NO_3_^–^ as well as the availabilities of soil TN and OM in the rhizosphere soil because of the N_2_-fixing functions of its roots [78]. The *Hippophae rhamnoides* mixed forests significantly increased the OM, TN, AP, and AK contents compared with the pure forests [79]. Different types (tree, shrub, and grass) and sources (artificial and natural) of vegetation (*R*. *pseudoacacia*, *Caragana korshinskii*, and grassland) strongly affected the soil AP, TN, NH_4_^+^-N, and pH levels [80]. We also found that the soils under natural vegetation (QW, PON, and BM) were accumulated more TN, OM, AN, and AK than the other vegetation cover types.

We found that the TN, TP, AN, AP, AK, and OM contents were highly variable in the 0–60 cm soil layers (CV ≥ 20%) due to complex topographical changes over a short scale in the study area, even within same land use type (S3 Fig). Therefore, this case limited the results to interpolate for a long distance. Terrain factors, including the relative elevation (*Hr*), slope (*β*), aspect (*A*), sin of aspect (*sinA*), and the relative position index (*RPI*) significantly affected the concentrations and spatial heterogeneity of the eight soil chemical properties (Table 8) because topography has important effects on the runoff potential and soil-forming in hilly landscapes [3]. Similar studies have also demonstrated that topographical factors, including elevation (H), slope (*β*), upslope contributing area (CA), terrain wetness index (*TWI*), and stream power index (*SPI*) have important effects on the redistribution of soil total carbon (TC), OM, N, and P across the landscape [3,24,35,80,81]. In conclusion, the TN, AN, AP and OM contents in 0–60 cm soil layers were significantly correlated with terrain factors (in bold and italic in Table 8), including *RPI*, *M*, *QFD*, and *TWI*.

We found that the TN, TP, AN, AP, AK, OM, and pH levels, and the soil fertility in Yan’an urban forest all decreased as the soil depth increased under various vegetation cover types (Figs 5 and 6), possibly due to differences in nutrient infiltration and absorption by soil colloids along the vertical profile [82,83], as well as variations in the microbial community [84] and litter [85]. The specific causal relationships require further investigation.

### Single soil fertility and comprehensive fertility at the urban forest scale

To the best of our knowledge, there is a lack of criteria for synthetically assessing the fertility status of forest soils. Previous studies of agricultural soil fertility assessments mainly on relatively stable properties rather than unstable properties, because that the former usually exhibit greater variation. However, in this case study, the CV values for the unstable properties (AN, AP and AK) were slightly larger than (0.39–1.74 fold) that of the corresponding those for the relatively stable properties (TN, TP, and TK), but, they all close to similar range (17%–91%) and trend (relatively stable among different soil layers). Which resulted from relatively homogeneous soil parent material (Quaternary loess) and most soils were free from anthropic intervention for ten years. Furthermore, some previous studies also used kriging interpolation methods to predict and assess the unstable soil properties, such as, AK (including, water-soluble potassium and exchangeable potassium) [26,86], extractable phosphorus (EP) [86]. According to the Liebig's law of the minimum, we referenced the soil fertility assessments for cropping lands in agricultural ecosystems, based on an improved Nemerow index, where we replaced the maximum value for a single-factor in the original Nemerow index, and an additional amendments (n –1)/n were used to increase the credibility of the evaluation. Relatively stable properties and unstable properties were both integrated into our comprehensive soil fertility index (F). The differential grading of each soil chemical property made them compatible. Our conclusions are similar to these obtained in related studies conducted in the Loess Plateau region of China. The soil OM contents decreased as the soil depth increased and they were insufficient in most areas with low vegetation cover because of scarce litter in the surface layer. Moreover, the AN was deficient in the 0–60 cm soil profile, especially at the margins of the study area with steep terrain because of more acute soil erosion due to surface runoff [41]. Furthermore, the soil AP in the 0–60 layer was at a low level in 90% of areas surveyed. The soil TN levels were below average in 66% areas of tested, especially in farmland. Chen et al. (2017) and Liu et al. (2013) had found that the soil C, TN, and TP levels on the Loess Plateau were lower compared with the whole of China [37,87]. We found that the soil AK contents of the 0–60 cm profile were at the average level in most areas (more than 60%). The soil TP and TK were both sufficient in the 0–60 cm layer. The soil pH was alkaline in the 0–60 cm depth, mostly in the range of 7.00–8.30. Hence, the AN, TN, and OM were the limiting factors in our study area, which were consistent with the previous studies [37,87].The soil comprehensive fertility index (F) was higher in the southwest of the study area compared with other areas, possibly due to differences in vegetation (mainly natural broad-leaved) and climate (precipitation and temperature, S4 Fig) [5]. In subsequent studies, the characteristics of soil nutrient requirements of specific target plants should be determined to obtain more accurate outputs.

## Conclusions

This case study based on the spatial mapping and the variations in the eight soil chemical properties (TN, TK, TP, AN, AP, AK, OM, and pH) as well as the soil fertility in the hilly gully Loess Plateau at the urban forest scale obtained three main findings. First, the majority of the soil chemical properties exhibited moderate spatial dependencies and they were suitable for ordinary kriging interpolation, whereas others with weak spatial dependencies required regression kriging interpolation with topographic factors (elevation, slope, aspect, the sin of aspect, relative position index, etc.) as auxiliary variables. Second, the concentrations of the eight soil chemical properties were significantly influenced by the vegetation cover types due to differences in TNPS and DBH at the sample-plot scale. Third, the AN, TN, and OM were the limiting factors in our study area, and which could be improved by natural broad-leaved forests (*Q*.*wutaishanica* forests, *Betula platyphylla* forests, etc.).

## Supporting information

S1 Table Overview of the 95 sample plots used in this study. (DOCX)

S2 Table Importance values (%) for tree species in the 95 sample plots. (DOCX)

S3 Table The optimized semivariograms parameters used in the OK and RK based on the modeling dataset. (DOCX)

S4 Table Pearson correlation coefficients for the eight SCPs of each soil layers and the total number of plant species (TNPS). (DOCX)

S5 Table Pearson correlation coefficients for the eight SCPs of each soil layers and the diameter at breast height (DBH). (DOCX)

S1 Fig Semivariograms of the raw or log-transformed data for the eight SCPs using the OK interpolation method. TN, TP, TK, AN, AP, AK, OM and pH are proxy for total nitrogen, total phosphorus, total potassium, available nitrogen, available phosphorus, available potassium and pH, respectively. The range data: 0–20, 20–40, 40–60 represents the depth of soil layer with 0–20 cm, 20–40 cm and 40–60 cm, respectively. These have the same meanings in the S2 Fig.
(PDF)

S2 Fig Semivariograms based on the interpolated residuals obtained by OLS for the eight SCPs using the RK interpolation method. (PDF)

S3 Fig Spatial distribution maps for the auxiliary terrain variables used in the RK interpolation method. (**a**) *Hr*, relative elevation; (**b**) *β*, slope; (**c**) *A*, aspect; (**d**) cosA; e) sinA; (**f**) *C*_*v*_, slope of slope; (**g**) *C*_*h*_, slope of aspect; (**h**) *QFD*, range of change in the elevation; (**i**) *M*, terrain roughness on the surface; (**j**) *RPI*, relative position index; (**k**) *TWI*, terrain wetness index; and (**l**) *SPI*, stream power index.
(PDF)

S4 Fig Spatial distribution maps of total annual precipitation (TAP) and mean annual air temperature (MAT) during 1951–2013. (PDF)
