# Supplementary material for: Effects of vegetation, terrain and soil layer depth on eight soil chemical properties and soil fertility based on hybrid methods at urban forest scale in a typical loess hilly region of China
Source: PLoS One. 2018 Oct 18;13(10):e0205661. doi: 10.1371/journal.pone.0205661 (PMC6193655; doi:10.1371/journal.pone.0205661)
Supplement: S1 Table — (DOCX) [file pone.0205661.s001.docx]

**S1 Table. Overview of the 95 sample plots used in this study.**

| **Smapl-e plot No.** | **Vegetati-on**  **cover type** | **DBH / cm^2^**  **(Mean ± SD)** | **Tree Species Composition / (the total number of )** **plant species** | **Smapl-e plot No.** | **Vegetati-on**  **cover type** | **DBH / cm^2^**  **(Mean ± SD)** | **Tree Species Composition /**  **(the total number of )** **plant species** |  |
| --- | --- | --- | --- | --- | --- | --- | --- | --- |
| 1 | QW | 733.99±2383.08 | 12QW + 9PO + 1AS /  (29) | 49 | BM | 132.53±95.35 | 21SR + 20PB + 6EM + 5AFl + 4RU + 1UP / (38) |  |
| 2 | POP | 136.00±105.30 | 29PO + 1PB / (19) | 50 | BM | 226.84±220.73 | 16PB + 15BP + 4SR + 3AFl + 3UP / (27) |  |
| 3 | SW | —— | HR / (12) | 51 | BM | 219.37±283.50 | 16BP + 10PD + 6AFl + 4EM + 1UP + 1SR  / (34) |  |
| 4 | GL | —— | TA, TV, MO / (10) | 52 | BM | 268.56±246.28 | 17BP + 10PD + 7AFl + 2EM + 1UP + 1SR / (34) |  |
| 5 | BM | 25.16±7.54 | 37AS + 1PB / (19) | 53 | BM | 193.21±149.77 | 36QW + 9PO / (17) |  |
| 6 | BM | 14.02±9.47 | 26PH + 4 RP / (18) | 54 | RP | 221.00±71.18 | 19RP / (12) |  |
| 7 | RP | 42.19±40.22 | 34 RP + 7 UP / (19) | 55 | PT | 164.84±132.79 | 26PTa / (10) |  |
| 8 | GL | —— | HA, PBi, SV, IP, etc. / (8) | 56 | QW | 216.24±142.16 | 39QW + 1PTa / (24) |  |
| 9 | QW | 202.08±374.39 | 13QW + 2 + AFl + 2 AS + 2 UP / (24) | 57 | QW | 192.67±158.35 | 28QW + 4PO + 3SR + AFl / (21) |  |
| 10 | PT | 172.06±112.33 | 39 PTa + 2 PD +1 PB + 1AFl / (31) | 58 | POP | 110.78±90.90 | 62PO + 9QW / (21) |  |
| 11 | RP | 285.02±371.18 | 32RP / (17) | 59 | QW | 277.57±216.35 | 21QW + 1PO + 1AFl + 1CP +1SR / (23) |  |
| 12 | RP | 84.98±43.52 | 55RP / (14) | 60 | QW | 277.69±272.34 | 37QW + 1AS / (11) |  |
| 13 | RP | 125.94±93.42 | 24RP / (22) | 61 | QW | 277.83±210.26 | 29QW + 4AB + 1AS |  |
| 14 | BM | 266.27±58.00 | 6PD + 4UM / (23) | 62 | QW | 1058.90±5314.77 | 42QW / (15) |  |
| 15 | QW | 193.29±161.84 | 30QW / (14) | 63 | CL | —— | Spring Corn / (1) |  |
| 16 | QW | 371.09±498.65 | 6QW / (16) | 64 | RP | 77.47±39.72 | 63RP / (21) |  |
| 17 | QW | 394.09±223.09 | 20QW / (15) | 65 | RP | 124.59±60.53 | 86RP / (19) |  |
| 18 | QW | 101.20±50.89 | 75QW + 1AS / (12) | 66 | RP | 488.19±249.62 | 34RP / (17) |  |
| 19 | BM | 427.06±267.36 | 41PD + 7 PU / (13) | 67 | RP | 258.34±234.55 | 17RP / (24) |  |
| 20 | BM | 113.80±107.27 | 20AB + 20 EM + 10 KP + 3UP / (20) | 68 | RP | 116.06±56.98 | 36RP / (27) |  |
| 21 | QW | 189.95±150.50 | 15QW + 1 AN / (19) | 69 | RP | 95.66±54.57 | 41RP / (19) |  |
| 22 | RP | 77.14±46.82 | 57RP + 2 AS / (22) | 70 | RP | 135.74±90.81 | 36RP / (20) |  |
| 23 | RP | 78.25±43.17 | 54RP / (19) / (22) | 71 | RP | 114.89±115.78 | 45RP / (30) |  |
| 24 | RP | 224.32±292.55 | 29RP + 13 UP + 1PD + 1PB / (24) | 72 | RP | 313.55±242.25 | 41RP / (26) |  |
| 25 | RP | 112.80±64.00 | 11RP / (17) | 73 | RP | 332.70±122.47 | 14RP / (18) |  |
| 26 | BM | 164.46±162.41 | 8PD + 7PTa / (20) | 74 | RP | 240.61±125.49 | 18RP / (25) |  |
| 27 | RP | 241.00±161.98 | 27RP / (19) / (24) | 75 | RP | 353.95±285.60 | 19RP / (23) |  |
| 28 | RP | 248.17±183.73 | 9RP + 4EM + 2PU + 1 XS / (20) | 76 | RP | 280.17±161.35 | 17RP / (27) |  |
| 29 | EF | 36.74±6.58 | 17MP / (8) | 77 | RP | 206.30±194.48 | 19RP / (23) |  |
| 30 | RP | 91.60±55.98 | 53RP / (23) | 78 | RP | 60.39±41.66 | 66RP / (23) |  |
| 31 | RP | 129.19±146.55 | 29RP / (18) | 79 | RP | 102.41±60.85 | 58RP / (20) |  |
| 32 | RP | 268.38±326.99 | 16RP / (17) | 80 | RP | 96.01±53.48 | 38RP / (22) |  |
| 33 | BM | 180.54±110.55 | 8PD + 7PTa / (13) | 81 | RP | 99.35±81.45 | 39RP / (25) |  |
| 34 | POP | 125.92±100.72 | 56PO + 1 UM + 1PB + 1AB + XS / (29) | 82 | RP | 215.81±161.21 | 29RP / (20) |  |
| 35 | QW | 291.82±340.91 | 19QW + 3AS + 1PO + 1AB / (24) | 83 | RP | 191.90±102.34 | 23RP / (31) |  |
| 36 | POP | 99.81±81.23 | 66PO + 4QW + 4AS+ 1KP / (17) | 84 | POP | 28.76±11.99 | 36PO / (31) |  |
| 37 | RP | 180.54±110.55 | 33RP / (13) | 85 | PON | 137.65±79.82 | 71PO / (26) |  |
| 38 | POP | 125.92±100.72 | 56PO + 1UM + 1PB + 1AB + XS / (12) | 86 | PON | 195.44±117.67 | 45PO / (17) |  |
| 39 | QW | 291.82±340.91 | 19QW + 3AS + 1AB + 1 PO / (14) | 87 | POP | 33.25±19.65 | 39PO / (20) |  |
| 40 | QW | 215.39±199.87 | 17QW + 12AFl + 1PD + 1SR / (13) | 88 | POP | 22.53±8.98 | 22PO / (19) |  |
| 41 | QW | 198.45±153.28 | 16QW + 13AFl / (14) | 89 | PON | 88.33±62.21 | 32PO / (18) |  |
| 42 | QW | 135.06±155.94 | 54QW + 19AS + 2PB + 1CP / (22) | 90 | PON | 211.60±127.45 | 38PO / (21) |  |
| 43 | QW | 174.25±107.26 | 41QW + 2CP / (12) | 91 | POP | 61.76±33.47 | 79PO / (19) |  |
| 44 | QW | 322.19±274.67 | 21QW + 2CP + 1AS / (20) | 92 | PON | 262.12±111.73 | 39PO / (17) |  |
| 45 | BM | 162.36±130.80 | 26PTa + 7PD + 6UP + 5AFl + 2SR + 1PB / (22) | 93 | PON | 201.41±150.17 | 43PO / (21) |  |
| 46 | BM | 469.36±552.77 | 8AB + 4PD + 2UP + 1BP / (12) | 94 | PON | 182.63±216.68 | 51PO / (15) |  |
| 47 | QW | 339.35±291.56 | 41QW + 2CP / (12) | 95 | PON | 163.72±249.42 | 38PO / (13) |  |
| 48 | BM | 303.38±788.33 | 39BP + 3AFl + 3PB + 1QW / (24) |  |  |  |  | |

Abbreviations

1) Vegetation cover types: BM, Broadleaved mixed near-natural forest (15); CL, Cultivated land (1); EF, Economic forest (1); GL, Grassland (2); PON, *Platycladus orientalis* near-natural forest (8); POP, *Platycladus orientalis* plantation forest (9); PT, *Pinus tabuliformis* plantation forest (2); QW, *Quercus wutaishansea* natural forest (21); RP, *Robinia pseudoacacia* plantation forest (35); SW, Shrubwood (1).

2) Scientific names of species coded using the first letters of the species and genus names (except for several tree species because of duplications). AB*: Acer buergerianum* Miq*.;* AFl*: Acer florinii*; AG: *Acer ginnala* Maxim. subsp. Ginnala; AJ: *Albizia julibrissin* Durazz; AM: *Acer mono* Maxim; AN*：Acer negundo L.*; AP: *Amygdalus persica* L.; AS: *Armeniaca sibirica* (L.) Lam.; AV: *Armeniaca vulgaris* Lam*.*; BP: *Betula platyphylla* Suk.; CD: *Cedrus deodara* (Roxb.) G. Don; CP: *Crataegus pinnatifida* var. *pinnatifida*; EM: *Euonymus maackii* Rupr.; FC: *Fraxinus chinensis* Roxb.; FS: *Forsythia suspensa* (Thunb.) Vahl f. *suspensa*; GB: *Ginkgo biloba* L.; HA: *Heteropappus altaicus* (Willd.) Novopokr.; HR: *Hippophae rhamnoides* L.; IP: *Ixeris polycephala* Cass.; JF: *Juniperus formosana* Hayata; JR: *Juglans regia* L.; KP: *Koelreuteria paniculata* Laxm.; MA: *Morus alba* L.; MO: *Melilotus officinalis* (L.) Pall; MP: *Malus pumila* Mill.; PA: *Picea asperata* Mast.; PB: *Pyrus betulifolia* Bunge; PBi: *Potentilla bifurca* L.; PC: *Pistacia chinensis* Bunge; PCa: *Prunus cerasifera* Ehrhar f. *atropurpurea* (Jacq.) Rehd.; PD: *Populus davidiana* Dode; PH: *Populus hopeiensis* Hu et Chow in Bull.; PO: *Platycladus orientalis* (L.) Franco; PPi: *Pinus pinea* L.; PS: *Pinus sylvestris* Linn. var. *mongolica* Litv.; PTa: *Pinus tabuliformis* Carr.; PU: *Pyrus ussuriensis* Maxim.; QW: *Quercus wutaishanica* Mary; RP: *Robinia pseudoacacia* Linn.; RU: *Rhamnus utilis* Decne.; SC: *Sabina chinensis* (L.) Ant.; SJ: *Sophora japonica* Linn.; SJp: *Sophora japonica* Linn. var. *japonica* f. *pendula* Hort.; SJv: *Sophora japonica* Linn. var. violacea Carr.; SM: *Salix matsudana* var. *matsudana* f. *pendula* Schneid.; SR: *Syringa reticulata* (Blume) Hara var. amurensis (Rupr.) Pringle; SV: *Setaria viridis* (L.); Beauv.TA: *Thlaspi arvense* L.; TV: *Tripolium vulgare* Nees; UM: *Ulmus macrocarpa* Hance; UP: *Ulmus pumila* L.; WS: *Wisteria sinensis* (Sims) Sweet.; XS: *Xanthoceras sorbifolium* Bunge.
