# Supplementary material for: Effects of vegetation, terrain and soil layer depth on eight soil chemical properties and soil fertility based on hybrid methods at urban forest scale in a typical loess hilly region of China
Source: PLoS One. 2018 Oct 18;13(10):e0205661. doi: 10.1371/journal.pone.0205661 (PMC6193655; doi:10.1371/journal.pone.0205661)
Supplement: S2 Table — (DOCX) [file pone.0205661.s002.docx]

**S2 Table. Importance values (%) for tree species in the 95 sample plots**.

| Order | Species | IV | Order | Species | IV |
| --- | --- | --- | --- | --- | --- |
| 1 | *Robinia pseudoacacia* | 37.25 | 13 | *Syringa reticulata* | 1.01 |
| 2 | *Quercus wutaishansea* | 20.74 | 14 | *Euonymus maackii* | 0.97 |
| 3 | *Platycladus orientalis* | 14.25 | 15 | *Populus hopeiensis* | 0.69 |
| 4 | *Populus davidiana* | 5.76 | 16 | *Koelreuteria paniculata* | 0.29 |
| 5 | *Armeniaca sibirica* | 4.30 | 17 | *Crataegus pinnatifida* | 0.27 |
| 6 | *Pinus tabuliformis* | 3.14 | 18 | *Pyrus ussuriensis* | 0.20 |
| 7 | *Ulmus pumila* | 2.42 | 19 | *Xanthoceras sorbifolium* | 0.09 |
| 8 | *Pyrus betulifolia* | 2.03 | 20 | *Rhamnus utilis* | 0.05 |
| 9 | *Betula platyphylla* | 2.03 | 21 | *Acer mono* | 0.05 |
| 10 | *Acer egundo* | 2.01 | 22 | *Euonymus maackii* | 0.02 |
| 11 | *Malus pumila* | 1.28 | 23 | *Ulmus macrocarpa* | 0.018 |
| 12 | *Acer uergerianum* | 1.13 | 24 | *Malus pumila* | 0.002 |

The importance value (IV) of a species is defined as the average of the relative density, relative frequency, and relative dominance of the species, which is calculated using the following equation:

$\text{IV}_{i}\text{=}\frac{1}{3}\text{（}\frac{N_{Si}}{\sum_{i=1}^{n} \frac{N_{Si}}{A_{i}}}+\frac{Nq_{i}}{n\times m}+\frac{BA_{i}}{\sum_{i=1}^{n} BA_{i}})$, (S1)

where $N_{Si}$ is the number of individuals belonging to species i, $A_{i}$ is the area of all sample units, *n* is the number of species in the domain (*n* = 31), $Nq_{i}$ is the number of quadrats containing species *i*, $m$is the total number of quadrats (*m* = 34), and $BA_{i}$ is the basal area of species *i*.
