# Supplementary material for: Effects of vegetation, terrain and soil layer depth on eight soil chemical properties and soil fertility based on hybrid methods at urban forest scale in a typical loess hilly region of China
Source: PLoS One. 2018 Oct 18;13(10):e0205661. doi: 10.1371/journal.pone.0205661 (PMC6193655; doi:10.1371/journal.pone.0205661)
Supplement: S3 Table — (DOCX) [file pone.0205661.s003.docx]

**S3 Table. The optimized semivariograms parameters used in the OK and RK based on the modeling dataset.**

|  |  | Ordinary Kriging (OK) | | | | | | Regression Kriging (RK) | | | | | |
| --- | --- | --- | --- | --- | --- | --- | --- | --- | --- | --- | --- | --- | --- |
| Soil chemical  properies | soil  layer  depth  (cm) | data type | models | Co | Co+C | N/S | range  (m) | data type^1^ | models | Co | Co+C | N/S | range  (m) |
| TN | 0-20 | ln | Exp | 0.0001 | 0.2442 | 0.9996 | 3690 | raw | Exp | 0.1136 | 0.2282 | 0.5022 | 13600 |
| TN | 20-40 | ln | Exp | 0.0364 | 0.1838 | 0.8020 | 13440 | raw | Exp | 0.0129 | 0.0717 | 0.8201 | 8840 |
| TN | 40-60 | raw | Sph | 0.0007 | 0.043 | 0.9837 | 2170 | raw | Sph | 0.0008 | 0.0756 | 0.9894 | 3330 |
| TP | 0-20 | ln | Gau | 0.0379 | 0.0858 | 0.5583 | 4210 | raw | Sph | 0.0132 | 0.0306 | 0.5703 | 11530 |
| TP | 20-40 | ln | Gau | 0.0372 | 0.1014 | 0.6331 | 3930 | raw | Sph | 3.0200 | 7.0500 | 0.5716 | 11010 |
| TP | 40-60 | raw | Sph | 0.0077 | 0.0335 | 0.7700 | 7590 | raw | Sph | 0.0137 | 0.0292 | 0.0531 | 8520 |
| TK | 0-20 | ln | Exp | 0.0078 | 0.0314 | 0.7518 | 7270 | raw | Exp | 1.8280 | 3.6570 | 0.5001 | 5290 |
| TK | 20-40 | ln | Sph | 0.0118 | 0.0237 | 0.5021 | 10630 | raw | Sph | 3.0200 | 7.0500 | 0.5716 | 11010 |
| TK | 40-60 | raw | Gau | 1.872 | 4.972 | 0.6235 | 8770 | raw | Sph | 1.7570 | 3.6320 | 0.5162 | 11040 |
| AN | 0-20 | ln | Exp | 0.0211 | 0.0621 | 0.66020 | 5260 | reci | Sph | 0.0162 | 0.1934 | 0.9162 | 12030 |
| AN | 20-40 | ln | Sph | 0.1380 | 0.5350 | 0.7421 | 6010 | raw | Sph | 0.7060 | 2.0650 | 0.6581 | 1690 |
| AN | 40-60 | ln | Exp | 0.2104 | 0.4448 | 0.5270 | 22450 | reci | Lin | 0.0228 | 0.0228 | 0 | 29879 |
| AP | 0-20 | ln | Lin | 0.2784 | 0.2784 | 0 | 29879 | raw | Sph | 0.0900 | 3.8960 | 0.9769 | 2520 |
| AP | 20-40 | ln | Exp | 0.3130 | 0.6780 | 0.5383 | 15530 | raw | Lin | 1.5507 | 1.5507 | 0 | 29879 |
| AP | 40-60 | ln | Exp | 0.1660 | 0.4622 | 0.6408 | 6900 | raw | Exp | 0.0950 | 1.0630 | 0.9106 | 5700 |
| AK | 0-20 | ln | Exp | 0.0052 | 0.1444 | 0.9640 | 1290 | reci | Sph | 0.0010 | 1.2000 | 0.9992 | 3460 |
| AK | 20-40 | ln | Lin | 0.2506 | 0.2506 | 0 | 2988 | reci | Lin | 0.2506 | 0.2506 | 0 | 29879 |
| AK | 40-60 | ln | Sph | 0.0171 | 0.1702 | 0.8995 | 1690 | reci | Gau | 679.0 | 1821.0 | 0.6271 | 42590 |
| OM | 0-20 | raw | Sph | 0.1021 | 0.3702 | 0.7242 | 12670 | raw | Gau | 31.0 | 123.0 | 0.7480 | 36360 |
| OM | 20-40 | raw | Exp | 0.2120 | 0.8870 | 0.7610 | 71100 | raw | Sph | 10.90 | 46.80 | 0.7671 | 69290 |
| OM | 40-60 | raw | Exp | 0.2690 | 0.797 | 0.6625 | 71100 | raw | Sph | 1.7570 | 3.6320 | 0.5162 | 11040 |
| pH | 0-20 | raw | Gau | 0.0087 | 0.0792 | 0.8902 | 5310 | raw | Gau | 0.0145 | 0.0750 | 0.8067 | 5520 |
| pH | 20-40 | raw | Gau | 0.0092 | 0.0795 | 0.8843 | 5560 | raw | Gau | 0.4310 | 2.5080 | 0.8281 | 3100 |
| pH | 40-60 | raw | Gau | 0.0056 | 0.0500 | 0.8880 | 5310 | raw | Sph | 0.0001 | 0.0468 | 0.9979 | 10470 |

Data type: ln, Napierian logarithm; reci, reciprocal; 1 the interpolated residual.

Model: Exp, Exponential; Gau, Gaussian; Lin, linear; Sph, Spherical.

Spatial dependency class: Random (N/S = 0); Strong spatial dependency (0< N/S < 0.25); Moderate spatial dependency (0.25 ≤ N/S ≤ 0.75); Weak spatial dependency (N/S > 0.75) [1].

**References**

1. Cambardella CA, Moorman TB, Novak JM, Parkin TB, Karlen DL, et al. (1994) Field-Scale Variability of Soil Properties in Central Iowa Soils. Soil Science Society of America Journal 58: 1501-1511. https://doi.org/ 10.2136/sssaj1994.03615995005800050033x
