# Supplementary material for: Effects of vegetation, terrain and soil layer depth on eight soil chemical properties and soil fertility based on hybrid methods at urban forest scale in a typical loess hilly region of China
Source: PLoS One. 2018 Oct 18;13(10):e0205661. doi: 10.1371/journal.pone.0205661 (PMC6193655; doi:10.1371/journal.pone.0205661)
Supplement: S4 Table — (DOCX) [file pone.0205661.s004.docx]

**S4 Table. Pearson correlation coefficients for the eight SCPs of each soil layers and the total number of plant species (TNPS).**

| Soil layer  depth | TN | TP | TK | AN | AP | AK | OM | pH |
| --- | --- | --- | --- | --- | --- | --- | --- | --- |
| 0-20 cm | 0.2020(ns) | -0.3655** | 0.2630* | 0.2314* | 0.0353(ns) | 0.1473(ns) | -0.0106(ns) | -0.0590(ns) |
| 20-40cm | 0.0465(ns) | -0.3587** | -0.3463** | 0.1945(ns) | 0.0841(ns) | -0.0593(ns) | -0.0846(ns) | -0.1105(ns) |
| 40-60cm | 0.2018(ns) | -0.3766** | 0.2123(ns) | 0.2314* | 0.0689(ns) | -0.1561(ns) | -0.0390(ns) | -0.1438(ns) |

**, * and ns, represents the significant level of Pearson correlation significant at 0.0, 0.05, and not significant, respectively.
