# Supplementary material for: Effects of vegetation, terrain and soil layer depth on eight soil chemical properties and soil fertility based on hybrid methods at urban forest scale in a typical loess hilly region of China
Source: PLoS One. 2018 Oct 18;13(10):e0205661. doi: 10.1371/journal.pone.0205661 (PMC6193655; doi:10.1371/journal.pone.0205661)
Supplement: S5 Table — (DOCX) [file pone.0205661.s005.docx]

**S5 Table. Pearson correlation coefficients for the eight SCPs of each soil layers and the diameter at breast height (DBH).**

| Soil layer  depth | TN | TP | TK | AN | AP | AK | OM | pH |
| --- | --- | --- | --- | --- | --- | --- | --- | --- |
| 0-20 cm | 0.3443* | -0.3680** | -0.0860(ns) | 0.6038** | -0.2554* | 0.3542** | 0.8251** | 0.4381** |
| 20-40cm | -0.0280(ns) | -0.3883** | 0.0272(ns) | 0.2914** | -0.2053(ns) | 0.3438* | 0.7481** | 0.4447** |
| 40-60cm | -0.1490(ns) | -0.3580* | -0.0206(ns) | 0.2241* | -0.2117 | 0.3314** | -0.0593(ns) | -0.0450(ns) |

**, * and ns, represents the significant level of Pearson correlation significant at 0.0, 0.05, and not significant, respectively.
