# Supplementary material for: Effects of vegetation, terrain and soil layer depth on eight soil chemical properties and soil fertility based on hybrid methods at urban forest scale in a typical loess hilly region of China
Source: PLoS One. 2018 Oct 18;13(10):e0205661. doi: 10.1371/journal.pone.0205661 (PMC6193655; doi:10.1371/journal.pone.0205661)
Supplement: S1 Fig — TN, TP, TK, AN, AP, AK, OM and pH are proxy for total nitrogen, total phosphorus, total potassium, available nitrogen, available phosphorus, available potassium and pH, respectively. The range data: 0–20, 20–40, 40–60 represents the depth of soil layer with 0–20 cm, 20–40 cm and 40–60 cm, respectively. These have the same meanings in the S2 Fig. (PDF) [file pone.0205661.s006.pdf]

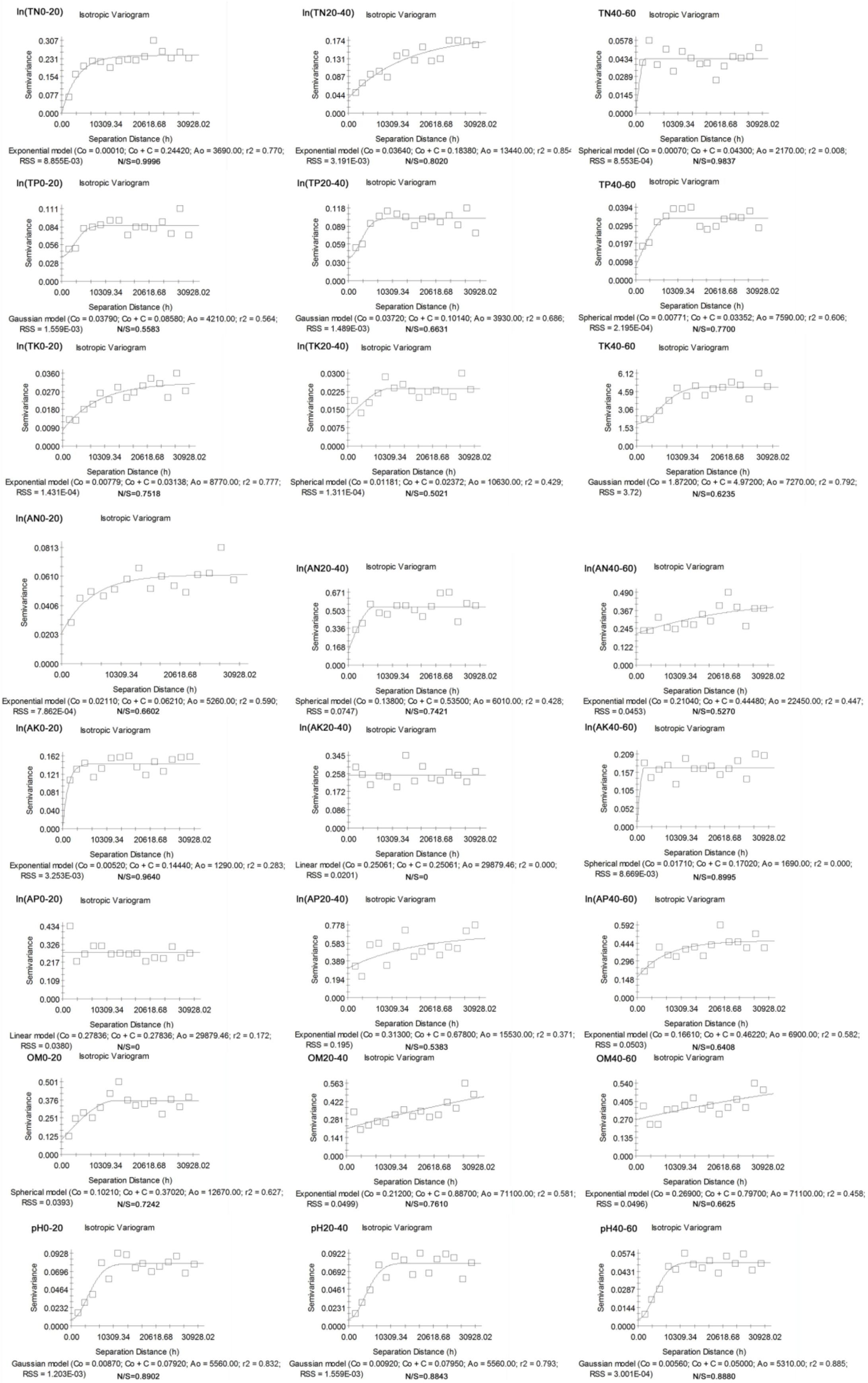

S1. Fig. Semivariograms of raw or log-transformed data for the eight SCPs using the OK interpolation method. TN, TP, TK, AN, AP, AK, OM and pH are proxy for total nitrogen, total

phosphorus, total potassium, available nitrogen, available phosphorus, available potassium and pH, respectively. The range data: 0-20, 20-40, 40-60 represents the depth of soil layer with 0-20 cm, 20-40 cm and 40-60 cm, respectively. These have the same meanings in the **S2 Fig.**
