# Supplementary material for: Effects of vegetation, terrain and soil layer depth on eight soil chemical properties and soil fertility based on hybrid methods at urban forest scale in a typical loess hilly region of China
Source: PLoS One. 2018 Oct 18;13(10):e0205661. doi: 10.1371/journal.pone.0205661 (PMC6193655; doi:10.1371/journal.pone.0205661)
Supplement: S2 Fig — (PDF) [file pone.0205661.s007.pdf]

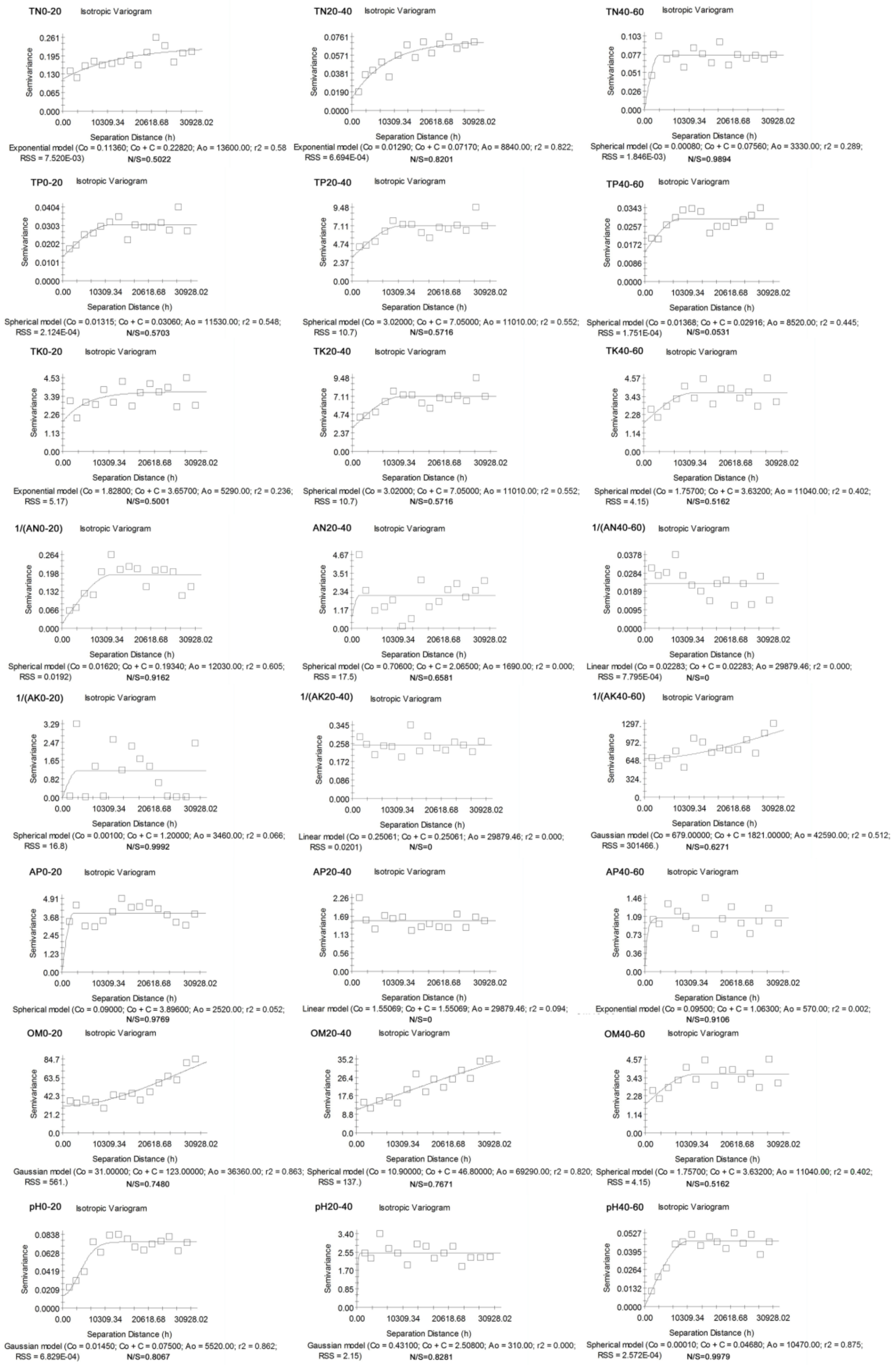

S2 Fig. Semivariograms based on the interpolated residuals obtained by OLS for the eight SCPs using the RK interpolation method.
