# Supplementary material for: Effects of vegetation, terrain and soil layer depth on eight soil chemical properties and soil fertility based on hybrid methods at urban forest scale in a typical loess hilly region of China
Source: PLoS One. 2018 Oct 18;13(10):e0205661. doi: 10.1371/journal.pone.0205661 (PMC6193655; doi:10.1371/journal.pone.0205661)
Supplement: S3 Fig — (a) Hr, relative elevation; (b) β, slope; (c) A, aspect; (d) cosA; e) sinA; (f) Cv, slope of slope; (g) Ch, slope of aspect; (h) QFD, range of change in the elevation; (i) M, terrain roughness on the surface; (j) RPI, relative position index; (k) TWI, terrain wetness index; and (l) SPI, stream power index. (PDF) [file pone.0205661.s008.pdf]

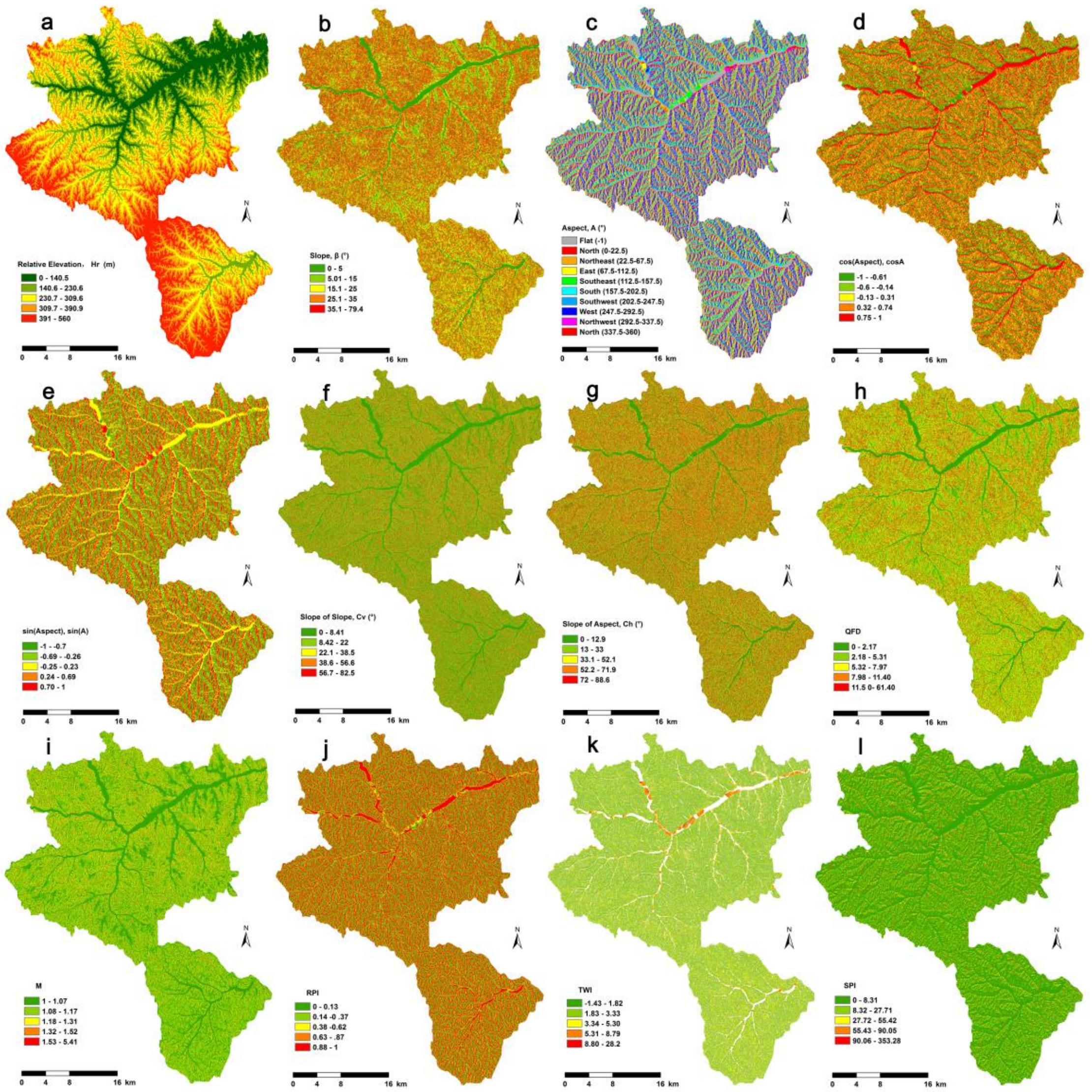

S3 Fig. **Spatial distribution maps for the auxiliary terrain variables used in the RK interpolation method.** (**a**) *Hr*, relative elevation; (**b**) *β*, slope; (**c**) A, aspect; (**d**) cosA; e) sinA; (**f**) C_v, slope of slope; (**g**) C_h, slope of aspect; (**h**) *QFD*, range of change in the elevation; (**i**) *M*, terrain roughness on the surface; (**j**) *RPI*, relative position index; (**k**) *TWI*, terrain wetness index; and (**l**) *SPI*, stream power index.
