# Supplementary material for: Effects of vegetation, terrain and soil layer depth on eight soil chemical properties and soil fertility based on hybrid methods at urban forest scale in a typical loess hilly region of China
Source: PLoS One. 2018 Oct 18;13(10):e0205661. doi: 10.1371/journal.pone.0205661 (PMC6193655; doi:10.1371/journal.pone.0205661)
Supplement: S4 Fig — (PDF) [file pone.0205661.s009.pdf]

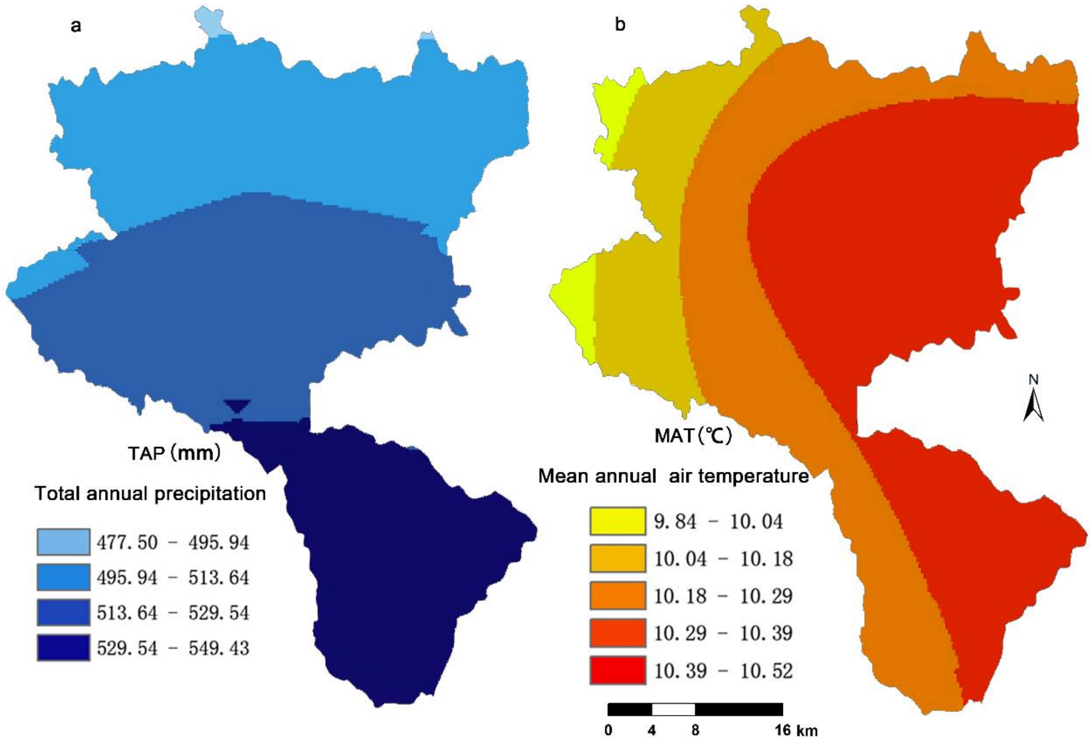

S4 Fig. Spatial distribution maps of total annual precipitation (TAP) and mean annual air temperature (MAT) during 1951-2013.
